# Molecular Mapping of Atherosclerotic Plaques with MALDI Mass Spectrometry Imaging

**DOI:** 10.3390/ijms27198745

**Published:** 2026-09-30

**Authors:** Dawid Leksa, Dorota Bartusik-Aebisher, Daniel Roshan Justin Raj, Angelika Myśliwiec, Alina Pietryszyn-Bilińska, Magdalena Moś, Piotr Oleś, Aleksandra Kawczyk-Krupka, David Aebisher

**Affiliations:** 1 Rzeszów Center for Vascular and Endovascular Surgery, 35-310 Rzeszów, Poland; dleksa@gmail.com; 2 Department of Biochemistry and General Chemistry, Faculty of Medicine, Collegium Medicum, University of Rzeszów, 35-310 Rzeszów, Poland; dbartusikaebisher@ur.edu.pl (D.B.-A.); amysliwiec@ur.edu.pl (A.M.); 3 English Division Science Club, Faculty of Medicine, Collegium Medicum, University of Rzeszów, 35-310 Rzeszów, Poland; dj135639@stud.ur.edu.pl; 4 Department of Internal Diseases, Angiology and Physical Medicine, Center for Laser Diagnostics and Therapy, Medical University of Silesia, Batorego 15, 41-902 Bytom, Poland; a.pietryszynbilinska@gmail.com (A.P.-B.); magdalenamos99@gmail.com (M.M.); piotroles@o2.pl (P.O.); akwczyk@gmail.com (A.K.-K.); 5 Department of Photomedicine and Physical Chemistry, Faculty of Medicine, Collegium Medicum, University of Rzeszów, 35-310 Rzeszów, Poland

**Keywords:** atherosclerosis, matrix-assisted laser desorption/ionisation (MALDI), mass spectrometry imaging (MSI), spatial molecular profiling, lipidomics, metabolomics, plaque vulnerability, biomarker discovery, targeted drug delivery, therapeutic monitoring

## Abstract

Atherosclerosis develops within heterogeneous arterial plaques. Here, even closely neighbouring regions can differ substantially in biological activity and potential vulnerability to rupture and thrombosis. Through matrix-assisted laser desorption/ionisation mass spectrometry (MALDI-MS) and especially MALDI imaging, these differences within the plaque can be studied while also preserving tissue architecture. With MALDI analysis, prepared tissue sections are converted into spatial molecular maps that show the differences in plaque chemistry across the arterial wall. These maps can be used to link changes in lipids and other biomolecules to regions affected by inflammation, necrosis, calcification and thrombosis. This narrative review, supported by a structured search of PubMed and PubMed Central from database inception to 14 September 2026, critically examines MALDI-based research in atherosclerosis across a translational continuum. The evidence base centres on spatial MALDI-MSI of plaque tissue, with non-imaging MALDI and orthogonal non-MALDI studies used as complementary context. Particular attention is given to the strength of evidence supporting proposed molecular signatures and to the distinction between exploratory findings and clinically validated applications. Integration of MALDI with histology, clinical imaging and emerging spatial-omics approaches could strengthen the biological interpretation of plaque-specific molecular signatures. However, methodological heterogeneity, incomplete molecular identification, small study populations and limited independent and prospective validation currently restrict translation into clinical practice.

## 1. Introduction and Methods

### 1.1. Introduction

Atherosclerosis is a chronic lipid-mediated inflammatory disease affecting medium- and large-sized arteries. It is the main pathological process behind heart issues such as myocardial infarction, ischaemic stroke and peripheral arterial disease [1]. The retention and modification of lipoproteins containing apolipoprotein-B in the arterial intima is the first step in atherosclerosis. It is then followed by endothelial activation, the recruitment of immune cells and maladaptive repair. Each plaque is not a uniform mass but a microenvironment on its own. Various scenarios can be seen in a single plaque of a few millimetres in size. For example, necrotic cores rich in lipids, inflammatory zones packed with macrophages, calcified deposits and fibrous caps may exist in very close proximity to each other [2]. Many factors can influence the growth and stability of plaques such as the phenotype switching of smooth muscle cells, extracellular-matrix remodelling, angiogenesis and several types of cell death [3]. This spatial heterogeneity can help to explain why similarly sized plaques can pose different levels of risk of rupture and thrombosis [4,5].

Conventional analytical methods have thus far provided only a partial view of plaque composition. Histology, for example, preserves tissue architecture and can identify regions of necrosis, fibrosis and calcification. Immunohistochemistry, on the other hand, can localise specific proteins or cell populations. However, staining-based methods usually depend on predefined targets and available antibodies, which limits their potential for untargeted molecular discovery [6]. Bulk transcriptomics show gene expression programmes, but transcript abundance itself cannot predict protein levels, post-translational modifications, or active metabolites [7]. Liquid-chromatography mass spectrometry can give sensitive molecular identification and quantification, but the homogenisation of tissues averages the signals across heterogeneous regions which leaves out information about their anatomical origin. These issues are important in atherosclerosis as the molecular meaning is closely related to the location and morphology of the plaque [8].

In this context, matrix-assisted laser desorption/ionisation mass spectrometry imaging (MALDI-MSI) provides a complementary analytical approach by preserving spatial molecular information that is lost when heterogeneous tissue is analysed in bulk. In MALDI-MS, analytes co-crystallised with a light-absorbing matrix are desorbed and ionised by a laser prior to mass analysis. MALDI-MSI extends this approach by acquiring a mass spectrum at defined x–y tissue coordinates and reconstructing the spatial distribution of selected ions as molecular maps without fluorescent or antibody labels [9]. Depending on sample preparation, MALDI-MSI can detect lipids, metabolites, peptides, proteins and also glycans within the histological framework [10]. Studies involving human and experimental plaques have identified atherosclerosis-associated lipids and demonstrated molecular differences between fibrous caps, necrotic cores and other histologically defined plaque regions [11,12]. In this way, MALDI-MSI can connect molecules to their precise anatomical location in the tissue, making it a useful tool for studying complex atherosclerotic plaques and identifying markers indicative of plaque growth and instability. Previous reviews have established the value of mass spectrometry imaging in vascular and cardiovascular research but have approached the field from different perspectives. Martin-Lorenzo et al. [13] reviewed the early application of MSI to arterial tissue and atherosclerosis, whereas Mezger et al. [14] considered MSI more broadly across cardiovascular diseases. More recently, Bookmeyer et al. [15] focused specifically on MALDI-MSI-based spatial lipidomics in atherosclerotic lesions, while Joshi et al. [16] examined mass-spectrometry-based spatial omics with emphasis on multimodal integration and computational analysis. Against this background, the purpose of the present review is not simply to catalogue molecular findings obtained using MALDI. Instead, we critically examine the translational progression of plaque-resolved MALDI research across lipids, metabolites, peptides, proteins and glycans, extending from plaque heterogeneity and vulnerability to biomarker discovery, multimodal integration, targeted drug delivery and treatment monitoring. Particular emphasis is placed on distinguishing exploratory molecular associations from findings that have undergone sufficient analytical, biological and clinical validation, and on identifying the methodological and evidential barriers that currently prevent translation into routine cardiovascular practice. The primary evidence domain considered in this review is spatial MALDI-MSI of plaque tissue. Non-imaging MALDI workflows and orthogonal non-MALDI studies are considered separately where they inform molecular identification, circulating biomarker discovery, biological validation or multimodal integration, rather than being treated as equivalent evidence of spatial MALDI-MSI performance.

### 1.2. Methods

This article was conducted as a narrative review supported by a structured literature search. The search was designed to identify a broad body of MALDI-based evidence relevant to atherosclerosis and to support a critical thematic synthesis, rather than to constitute a systematic or scoping review. A structured literature search was conducted in PubMed and PubMed Central (PMC), with the initial search performed on 22 June 2026 and an updated search conducted on 14 September 2026. Because PMC substantially overlaps with PubMed, it was used as a supplementary full-text source to identify potentially relevant publications not retrieved through the PubMed search; overlapping publications were considered only once during literature evaluation. Records published from database inception through 14 September 2026 were eligible for retrieval. Search terms relating to matrix-assisted laser desorption/ionisation and mass spectrometry imaging were combined with terms relating to atherosclerosis and atherosclerotic plaques using Boolean operators. The complete database-specific search strategies are shown in Supplementary Table S1.

Publications were considered relevant when they reported MALDI-based analyses related to atherosclerosis, including molecular mapping of atherosclerotic plaques, plaque-associated molecular signatures, biomarker discovery, treatment response or spatial assessment of therapeutic agents. Publications were not incorporated when atherosclerosis was not a primary disease context, when no MALDI-derived molecular-mapping, biomarker or treatment-response data relevant to the review were reported, when methodological or results reporting was insufficient to permit interpretation of the MALDI findings, or when the publication type was not relevant to the scope of the review. The searches were restricted to English-language publications. Grey literature was not systematically searched, and conference abstracts without a full peer-reviewed article were not included.

Following the removal of duplicate records, titles and abstracts were screened according to the eligibility criteria described above. Records were excluded at this stage if they focused on unrelated diseases or non-vascular tissues, or described purely methodological developments without a relevant atherosclerosis application. Potentially relevant publications subsequently underwent full-text assessment to confirm the presence of applicable MALDI-derived findings and sufficient methodological reporting. Publications addressing general MALDI methodologies or providing complementary biological evidence were considered separately as contextual literature rather than primary MALDI evidence. The literature identification and selection process is illustrated in Supplementary Figure S1.

The search strategy was intentionally broad because MALDI-based vascular studies are inconsistently indexed and may be described using broader terminology relating to arterial tissue, vascular plaques, cardiovascular disease, lipidomics, proteomics or mass spectrometry imaging. Potentially relevant publications were therefore evaluated for their relevance to the methodological and translational themes addressed in this review and synthesised narratively. No formal risk-of-bias assessment, quantitative meta-analysis or scoping-review data-charting framework was applied. During synthesis, evidence was interpreted according to analytical modality (spatial MALDI-MSI, non-imaging MALDI-MS or orthogonal non-MALDI methods), sample type, experimental model (human, animal, ex vivo/artificial) and intended application. Studies without MALDI-derived data were used only as complementary mechanistic, validation or multimodal context and were not treated as direct MALDI-MSI evidence. Study-level characteristics of the primary MALDI evidence, including sample size, species, arterial bed, plaque phenotype, analytical workflow, analyte class, molecular-identification confidence, principal findings and study-specific limitations, are summarised in Supplementary Table S2.

## 2. Molecular and Pathophysiological Basis of Atherosclerosis

### 2.1. Spatial Origins of Plaque Heterogeneity

Atherosclerotic plaques arise preferentially at arterial sites exposed to disturbed flow, where endothelial activation promotes lipoprotein entry and retention. Retained apolipoprotein-B-containing particles undergo oxidative and enzymatic modification, while chemokine and adhesion-molecule signalling recruits monocytes that differentiate into macrophages. Uptake of modified lipoproteins produces lipid-laden foam cells and amplifies local inflammation [17,18,19,20,21,22,23,24,25,26]. For MALDI-MSI, the key consequence of these processes is spatial heterogeneity: lipid-rich foam-cell regions, inflammatory areas, vascular smooth-muscle-cell compartments and relatively preserved arterial wall may coexist within the same lesion. These chemically distinct microenvironments provide the anatomical basis for region-resolved molecular mapping rather than bulk averaging.

### 2.2. Plaque Progression and Vulnerability

**Figure 1:**
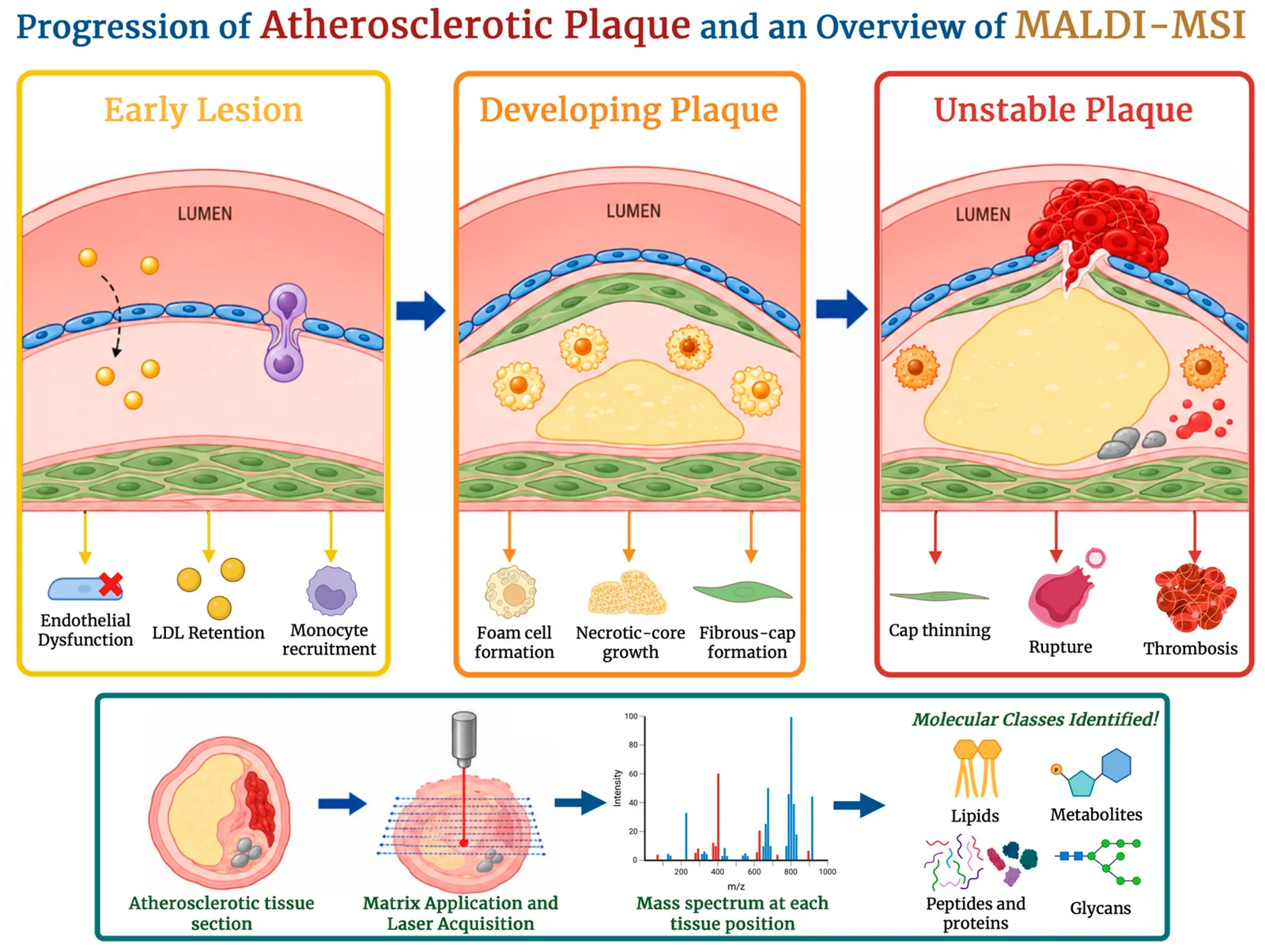
Overview of the progression of atherosclerotic plaques and the MALDI-based analysis of lipids, metabolites, peptides, proteins, and glycans within the plaque tissue. Created in BioRender. Roshan, D. (2026) https://BioRender.com/770ukss (accessed on 25 August 2026).

With plaque progression, persistent lipid loading, cell death, defective efferocytosis and cholesterol-crystal-associated inflammatory signalling contribute to expansion of the necrotic core. In parallel, inflammatory proteolysis and loss of matrix-producing smooth-muscle cells can weaken the fibrous cap, while intraplaque haemorrhage, calcification and repeated rupture and healing further increase structural and molecular heterogeneity. Advanced lesions may ultimately undergo cap rupture or endothelial erosion, exposing thrombogenic material and promoting platelet activation and thrombosis [27,28,29,30,31,32,33,34,35,36,37,38,39,40,41,42,43,44]. From the perspective of MALDI-MSI, these processes are important because fibrous caps, necrotic cores, macrophage-rich shoulders, calcified areas and haemorrhagic or thrombotic regions can be analysed as separate regions of interest. Molecular signals can therefore be interpreted according to their histological location rather than as averages across the entire plaque. Figure 1 summarises these major stages of plaque progression and the molecular classes that can be mapped by MALDI.

## 3. MALDI-Based Technologies for Atherosclerosis Research

### 3.1. How MALDI Creates Molecular Maps

MALDI is a soft ionisation technique that generally produces limited fragmentation, allowing many biomolecules to be transferred into the gas phase as intact ions [45]. The sample itself is either mixed or coated with an excess of matrix that co-crystallises with the analytes and absorbs the laser wavelength [46]. When a pulsed laser irradiates the crystals, the matrix becomes excited and produces a plume that contains the desorbed matrix and analyte molecules. Within this plume of matrix and analyte, proton transfer, deprotonation or cation attachment will generate charged analytes [47]. After the ions have been accelerated by an electric field, they are then separated according to their mass-to-charge ratio (*m*/*z*). Generally, lighter ions have been known to reach the detector before heavier ions in MALDI time-of-flight (MALDI-TOF) instruments. Their arrival times are converted into *m*/*z* and the responses from the detector will determine peak intensities and produce a mass spectrum [46]. While a reflectron TOF can give better mass resolution, MALDI-TOF/TOF instruments first select a precursor ion, then fragment it and analyse the products during a second TOF stage which helps with molecular identification [48].

MALDI mass spectrometry imaging (MALDI-MSI) is able to retain the spatial distribution of molecules within tissues. A thin section is first placed on a conductive slide which is then evenly coated with matrix and scanned by the laser in a raster pattern [49]. At every *x*–*y* coordinate, a mass spectrum is recorded so each sample position now becomes a molecular pixel [50]. Through software, the intensity of a chosen *m*/*z* signal is extracted across all coordinates, and an ion map is reconstructed showing its distribution in tissue [51]. Therefore, without the help of antibodies, fluorescent probes or other labels, hundreds of lipids, metabolites, peptides, proteins or glycans can be mapped together [9]. This approach enables molecular differences between histologically similar plaque regions to be investigated [11].

Sample preparation should be adapted to the molecular class being investigated. Fresh-frozen arterial tissue is generally preferred for native lipid and metabolite imaging because it minimises formalin-induced chemical modification and avoids the extraction of solvent-soluble analytes associated with paraffin processing [11,52,53]. In human carotid plaques, cryosections have been vacuum-dried and directly coated with DHB or norharmane without a pre-imaging washing step [11]. By contrast, protein and peptide imaging may benefit from controlled washing to reduce salts and abundant lipids that contribute to ion suppression. Martin-Lorenzo et al. directly compared five preparation protocols in healthy and atherosclerotic human arteries and demonstrated an optimised workflow for protein MALDI-MSI at 30 µm spatial resolution [54]. FFPE workflows may additionally use sequential alcohol- or Carnoy-based washing before antigen retrieval and enzymatic digestion [53].

Fixation therefore changes the molecular classes that can be effectively analysed. Fresh-frozen tissue is generally preferable for native lipids and small metabolites, whereas formalin fixation causes protein cross-linking and paraffin processing removes many lipid and other solvent-soluble species. MALDI can additionally analyse plasma or serum, isolated lipoproteins and secretomes from cultured vascular cells [55,56,57]. FFPE tissue consequently requires deparaffinisation and antigen retrieval and is commonly analysed after on-tissue digestion: trypsin releases peptides for proteomic imaging [58], whereas peptide-N-glycosidase F (PNGase F) releases N-linked glycans while preserving their spatial distribution [59,60].

Matrix selection is strongly dependent on the molecular class being investigated and the ionisation polarity and can substantially influence analyte coverage and apparent spatial contrast. For lipid imaging, 2,5-dihydroxybenzoic acid (DHB) is widely used, particularly in positive-ion mode, whereas 9-aminoacridine (9-AA) is particularly suited to negative-ion detection of acidic phospholipids and other negatively charged lipid species [59]. In human carotid atherosclerotic plaques, DHB and norharmane have been directly compared for spatial lipid imaging; DHB produced a significantly greater number of detected lipid ions than norharmane, while the use of both matrices provided complementary information and helped to assess matrix-dependent ionisation bias [11]. α-Cyano-4-hydroxycinnamic acid (CHCA), in contrast, is commonly used after on-tissue enzymatic digestion for peptide imaging and can also support the detection of N-glycans released by peptide-N-glycosidase F (PNGase F) [58,60]. Matrix selection therefore determines not only signal intensity but also which molecular classes and ion species are preferentially represented in the resulting image. Matrix deposition is similarly important because crystal size, coating homogeneity and solvent composition influence analyte extraction, ionisation efficiency and spatial fidelity; controlled spraying or sublimation can produce more homogeneous coatings, whereas excessive solvent exposure may promote analyte delocalisation [58,59]. The principal applications, ionisation polarities, advantages and limitations of commonly used MALDI-MSI matrices are summarised in Table 1.

### 3.2. From Mass Signals to Molecular Identity

An *m*/*z* signal alone is generally insufficient for confident molecular identification, and additional evidence is often required. Theoretical masses and databases such as LIPID MAPS, METASPACE, MSiMass List or protein libraries can be used to compare against the mass measurements given by high-resolution instruments. Spatial filtering and database searching in atherosclerotic aorta have helped to identify lipids and proteins in the intima, media and calcified regions [61]. By isolating a particular precursor ion and comparing its fragments with reference spectra or predicted structures, tandem mass spectrometry aims to improve identification [62]. Findings from MALDI are often compared with liquid chromatography-tandem mass spectrometry of tissue extracts or microdissected regions because direct on-tissue fragmentation can have reduced sensitivity. While MALDI has the advantage of preserving the anatomical location, liquid chromatography-tandem mass spectrometry (LC-MS/MS) can provide much deeper coverage and better sequence or structural evidence. Combining these approaches can improve molecular annotation and validation [63]. Accordingly, metabolite assignments in this review are distinguished according to identification confidence: Metabolomics Standard Initiatives Level 1 requires confirmation against an authentic reference standard using orthogonal evidence, whereas Level 2 represents a putative molecular annotation based on spectral or database evidence [64]. Processing usually includes spectral alignment, baseline correction, normalisation, peak picking, multivariate analysis and spectral clustering. Importantly, these analytical choices can influence the apparent differences observed between tissue regions and are considered further in Section 7.1. Co-registration of MALDI images with hematoxylin and eosin staining, lipid staining, immunohistochemistry or calcification staining can then help localise molecular signals to defined regions of the plaque and arterial wall [65].

**Figure 2:**
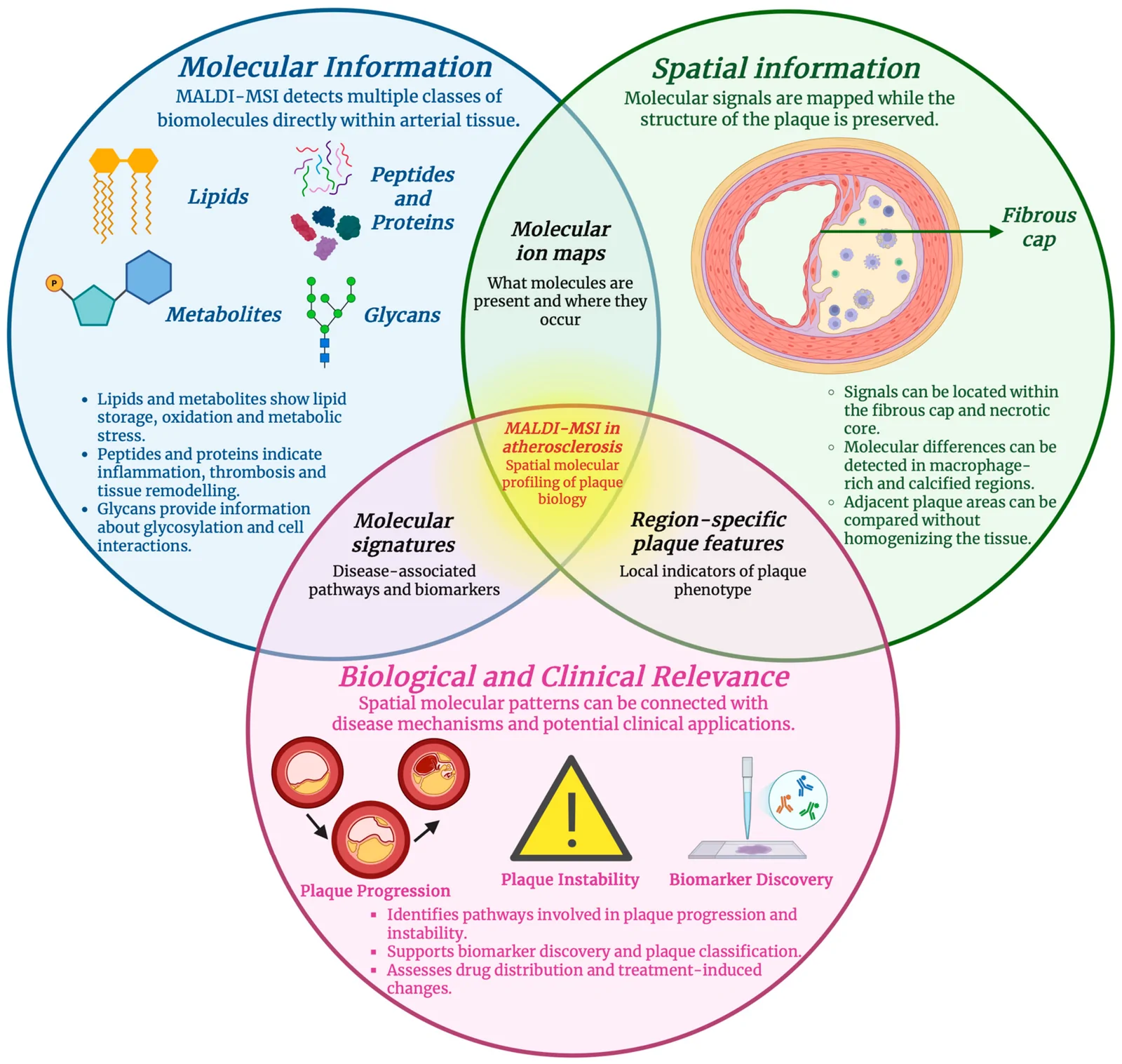
The Venn diagram shows the integration of molecular composition with anatomical location, which produces plaque signatures specific to each region. This combination supports the study of the mechanisms, biomarkers and treatment responses of atherosclerosis. Created in BioRender. Roshan, D. (2026) https://BioRender.com/rl6j26a (accessed on 25 August 2026).

To resolve molecular differences at cellular or subcellular scales, high-resolution MALDI imaging uses smaller laser spots and high-performance analysers. For example, atmospheric-pressure MALDI Orbitrap imaging has achieved micrometre-scale spatial resolution with high mass accuracy [66]. MALDI-2 is able to increase sensitivity for many lipids, metabolites and drugs as it utilises a second laser that post-ionises neutral molecules within the desorption plume [67]. Targeted methods such as MALDI immunohistochemistry use mass-tagged affinity probes to locate desired proteins and measure many other targets within the same tissue section [68]. With three-dimensional MALDI-MSI, which reconstructs molecular volumes from aligned serial sections, plaque components can be followed even through the vessel wall [69]. Through these various methods, MALDI-MSI can integrate anatomical location and biological relevance in atherosclerotic plaques as seen in Figure 2. The principles, sample types, applications and key considerations of the main MALDI-based methods in atherosclerosis are summarised in Table 2.

## 4. MALDI-Based Molecular Mapping of Atherosclerotic Disease

### 4.1. Lipid and Metabolic Changes in Plaques

MALDI-MSI can be used to map the distribution of lipids across the fibrous cap, macrophage-rich areas, vascular smooth-muscle-cell regions, necrotic core and calcified tissue. Human carotid studies have found cholesterol-related signals, cholesterol esters, phosphatidylcholines and sphingomyelins which show different lipid patterns between plaque compartments and also between symptomatic and asymptomatic disease [11,70]. It was observed that in the symptomatic plaques, the regions rich in macrophages were abundant in sphingomyelins and the region with intimal smooth muscle cells contained cholesterol and cholesterol esters [11]. Ceramides and lysophospholipids specific to plaques such as lysophosphatidylcholines, lysophosphatidic acids, lysophosphatidylethanolamines and lysophosphatidylinositols were mapped through human and animal datasets [70,71,72]. More recently, Ntshangase et al. identified region-specific lipid profiles in human carotid and rabbit atherosclerotic plaques, with alterations in sphingolipid and glycerophospholipid metabolism shared across species [73]. These spatial lipid patterns are consistent with established biological processes involving the retention and oxidative modification of apolipoprotein-B-containing lipoproteins within the arterial wall. Subsequent uptake of modified lipoproteins by macrophages and vascular smooth-muscle cells, cholesterol esterification and storage in cytoplasmic lipid droplets contribute to foam-cell formation and expansion of the lipid-rich plaque core [74,75]. These mechanisms provide biological context for interpreting the spatial lipid distributions identified by MALDI-MSI rather than mechanisms directly demonstrated by the MSI studies themselves.

MALDI-MSI can also map metabolite-associated signals and identify spatial patterns consistent with differences in metabolic pathways within plaques. In a study of nine human atherosclerotic plaques (five stable and four unstable), 170 of 855 detected peaks were annotated using database-assisted analysis, with 12 metabolites receiving additional MS/MS comparison against authentic standards [5]. Unstable plaques showed greater representation of ROS-related pathways, whereas stable plaques were more strongly associated with long-chain-fatty-acid metabolism [5]. These spatial findings can be interpreted in the context of cell-specific metabolic programmes described in complementary mechanistic studies. Macrophage activation is associated with increased glycolysis and alterations in oxidative phosphorylation, tricarboxylic-acid-cycle activity and fatty-acid metabolism [76]. Glycolytic metabolism also influences endothelial-cell function, while proliferating or osteogenic vascular smooth-muscle cells exhibit changes in glycolysis, glutamine utilisation and mitochondrial function [77,78,79]. Oxidative stress and lipid peroxidation may further contribute to inflammatory and plaque-destabilising processes [80,81]. These mechanisms provide biological context for the MALDI-MSI findings but were not directly demonstrated by the plaque MSI studies themselves.

### 4.2. Proteins, Peptides and Glycans in Plaques

Protein and peptide evidence should be distinguished according to whether MALDI was used for spatial imaging or for non-imaging molecular identification. MALDI-MSI can map intact proteins, peptides and on-tissue digestion products while preserving their location within arterial tissue [13]. By contrast, findings such as increased apolipoprotein E, clusterin and neutrophil defensin 1 in carotid atherosclerotic plaque secretomes [82], nitrated apolipoprotein A-I [83], cross-species plaque proteomic signatures [84], complement accumulation [85] and fibrin/fibrinogen products [86] derive from non-imaging or orthogonal analyses. These findings provide biological context and candidate targets for spatial validation but should not themselves be interpreted as region-resolved MALDI-MSI evidence.

Spatial glycomic evidence in atherosclerotic plaque remains less developed. MALDI workflows using peptide-N-glycosidase F can map released N-glycans in tissue and combine glycan with tryptic-peptide imaging [60], whereas much of the biological evidence linking glycosylation to atherosclerosis derives from non-MALDI studies [87,88,89]. These findings therefore provide biological context for future spatial validation rather than direct plaque-resolved MALDI glycomic evidence.

### 4.3. Molecular Signs of Plaque Vulnerability

Atherosclerotic plaques are mosaics of fibrous tissue, lipid-rich necrotic regions, inflammatory infiltrates, intraplaque haemorrhage, calcification and relatively preserved arterial wall. MALDI addresses this complexity by acquiring spectra across intact tissue that can subsequently be aligned with histology, allowing molecular signals to be compared between defined regions rather than averaged across the entire plaque. Region-specific analysis of human carotid plaques distinguished intraplaque haemorrhage, lipid-rich necrotic regions, intimal and medial smooth-muscle-cell regions, and collagen-rich from macrophage-rich regions [11]. Lipid and protein maps also distinguished the intima, media and calcified regions from less extensively diseased arterial tissue [61]. Distinct lipid patterns were associated with necrotic cores, foam-cell-rich areas and fibrous tissue in large-scale carotid imaging [70]. Consequently, regions such as the fibrous cap, macrophage-rich plaque shoulders, intraplaque haemorrhage and relatively unaffected tissue can be selected as regions of interest and compared with the necrotic core. Three-dimensional MALDI imaging can additionally determine whether region-specific molecular patterns persist across adjacent plaque sections [12].

Plaque phenotype terminology is retained according to the definitions used in the original studies. Symptomatic/asymptomatic status refers to clinical presentation, whereas stable/unstable generally reflects histopathological or experimental classification. These terms are therefore not treated as interchangeable. Spatial comparisons suggest that combinations of molecular changes may be associated with study-defined plaque phenotypes rather than a single universal marker. MALDI-MSI was able to reveal the regional differences present throughout the fibrous cap and necrotic core. In the same nine-plaque cohort, in which type V lesions were classified as stable or unstable according to fibrous-cap thickness (>200 μm and <200 μm, respectively), signals annotated as homoarginine were enriched in stable plaques, whereas 2-nonenal-associated signals were enriched in unstable plaques [5]. However, the study did not include an independent validation cohort. In a separate carotid cohort, symptomatic status was defined by transient ischaemic attack symptoms within six months before surgery (n = 9), while 20 patients were classified as asymptomatic [4]. Symptomatic plaques showed macrophage-rich regions enriched in phosphatidylcholines, whereas asymptomatic plaques showed greater polyunsaturated cholesteryl esters and triglycerides in macrophage-rich regions and lysophosphatidylcholines and cholesteryl esters in fibrous-cap smooth-muscle-cell regions [4]. These findings represent associations with clinical symptomatic status rather than direct evidence of histological instability or future rupture. Other MALDI studies have found extensive, tissue specific lipid signatures in advanced carotid and coronary plaques [52,72]. Taken together, these findings support the biological plausibility of region-specific molecular signatures of plaque instability, but they do not yet establish a clinically transferable vulnerability signature. Most available studies involve relatively small cohorts and cross-sectional analysis of excised plaques, while analytical workflows and definitions of plaque stability differ between studies. Moreover, few candidate signatures have undergone independent replication or prospective evaluation against subsequent cardiovascular events. Multivariate analysis provides a potential route from individual molecular markers to composite plaque signatures. Orthogonal partial least-squares discriminant analysis (OPLS-DA) has already been applied to human carotid MALDI-MSI data to identify lipid profiles characteristic of defined plaque components [70], demonstrating that spatial spectral patterns can be classified rather than interpreted one molecule at a time. More broadly, supervised approaches such as PLS-DA and random forest classifiers have been applied to MSI datasets for tissue classification and feature selection [90,91]. In atherosclerosis, similar approaches could integrate multiple region-specific signals into composite vulnerability models, although patient-level validation in independent cohorts would be required before such scores could be considered predictive. MALDI-MSI should therefore currently be regarded as a discovery and mechanistic stratification tool for plaque vulnerability rather than a validated predictor of plaque rupture or thrombosis. Table 3 provides a molecular-level synthesis of representative findings. Detailed study-level characteristics of the supporting MALDI evidence are provided in Supplementary Table S2.

## 5. Biomarker Discovery and Translational Validation

Biomarker translation comprises distinct stages. Here, biomarker discovery refers to identification of candidate molecular signatures; analytical validation to demonstration of reproducible measurement; clinical validation to confirmation of diagnostic or prognostic performance in independent patient cohorts; and clinical utility to evidence that biomarker use improves clinical decision-making or outcomes. Most plaque MALDI signatures discussed below remain at the discovery stage.

### 5.1. Plaque Tissue Biomarker Discovery

Plaque-tissue MALDI-MSI can generate candidate biomarker signatures by combining molecular composition with histological location. As detailed in Section 4.3, region-specific lipid and metabolite patterns have been associated with study-defined symptomatic/asymptomatic and stable/unstable plaque phenotypes [4,5,11]. From a translational perspective, however, these findings currently represent biomarker-discovery signals rather than validated clinical classifiers. Reproducible analytical workflows, independent validation cohorts and prospective evaluation against cardiovascular outcomes will be required before such signatures can be considered clinically applicable [92].

### 5.2. Circulating Biomarker Discovery

Spatial plaque signatures should therefore be regarded as sources of candidate circulating biomarkers rather than direct surrogates for blood concentrations. A molecule enriched within a necrotic core or inflammatory plaque region must first reach the circulation through secretion, tissue injury, cell death or extracellular-vesicle transport, after which it is diluted and may undergo proteolytic degradation or hepatic and renal clearance. Consequently, tissue enrichment does not necessarily translate into a measurable or plaque-specific change in plasma, and candidate markers identified by MALDI-MSI require validation in matched blood samples.

Serum, plasma, lipoproteins, extracellular vesicles and circulating peptides or lipids can be analysed through MALDI profiling. These non-imaging workflows are analytically distinct from plaque MALDI-MSI and require separate pre-analytical standardisation, analytical validation and independent clinical validation. The MALDI-TOF analysis of serum N-glycans was able to identify marker panels related to the severity of coronary artery narrowing. Plasma proteomics, alternatively, found the proteins commonly associated with coronary artery disease and prognosis in peripheral arterial disease [95,96]. MALDI-TOF has been used to characterise the lipid composition of isolated low-density lipoprotein (LDL) and high-density lipoprotein (HDL) particles [97] and to assess oxidative modifications of apolipoproteins that may reflect lipoprotein dysfunction [98]. Extracellular vesicles released by platelets in diabetes were analysed by nanoflow liquid chromatography coupled with MALDI-MS/MS (nanoLC-MALDI-MS/MS). The findings report that these vesicles protect and carry molecules that provide information about the cells that they originated from [99]. Minimally invasive blood sampling remains attractive for screening, risk assessment and longitudinal monitoring, but translation from tissue to circulation presents substantial analytical challenges. The plasma proteome spans an exceptionally wide concentration range, and highly abundant proteins can suppress detection of low-abundance, tissue-derived molecules, particularly when candidate markers occur at low or nanomolar concentrations [100]. Circulating concentrations are also shaped by proteolytic degradation, hepatic and renal clearance, systemic inflammation and pre-analytical factors, meaning that a signal detected in blood may not specifically reflect plaque abundance [101]. Depletion, fractionation or targeted enrichment may therefore be required, followed by validation in independent cohorts before plaque-derived MALDI signatures can be considered clinically useful circulating biomarkers.

### 5.3. Linking Molecular Profiles to Clinical Risk

Evidence linking MALDI-derived molecular profiles to clinical risk comes from distinct analytical domains and should therefore be interpreted separately. When plasma proteomics using two-dimensional difference gel electrophoresis (2D-DIGE) and MALDI-TOF was applied to peripheral arterial occlusive disease, it identified transthyretin and complement factor B as candidate proteins associated with favourable or poor prognosis [96]. Haptoglobin has been identified by MALDI-based plasma proteomics as a potential prognostic marker after acute myocardial infarction [102]. Also, serum protein fingerprints were associated with 30-day and three-year cardiovascular events in acute coronary syndromes [103]. Tissue MALDI-MSI can map lipid patterns associated with necrotic-core, foam-cell and fibrin-rich plaque regions [70]. Neointimal diacylglycerols and lysophosphatidylcholines had changed after angioplasty in experimental restenosis [104]. Collectively, however, these findings remain exploratory rather than clinically validated. The key translational question is no longer whether MALDI can identify molecular differences associated with cardiovascular disease, but whether these signatures provide reproducible and clinically meaningful information beyond established risk factors and imaging approaches. This will require external validation in independent, adequately powered cohorts with predefined cardiovascular endpoints and assessment of incremental discrimination, calibration and clinical utility beyond established clinical and imaging predictors. Until such evidence is available, MALDI-derived tissue and circulating signatures should be considered candidate biomarkers rather than clinically actionable risk-prediction tools.

A potential route towards clinical translation is integration of MALDI signatures with plaque location, burden and biological activity. Angiography shows the level of luminal stenosis while ultrasound examines the structure of the artery. Computed tomography (CT) can measure calcium and plaque composition, whereas positron emission tomography (PET) can detect inflammation and small calcium deposits. The shape and structure of the vessel wall can be seen through magnetic resonance imaging (MRI) [105]. MALDI-MSI can then show which molecules are related to those imaging features. This strategy can be seen in its ability to co-localise an atherosclerosis-targeted MRI with extracellular-matrix-rich plaque [106]. Using spatial lipidomics alongside genomics, transcriptomics and conventional proteomics can explain how inherited risk and gene expression can play a part in the molecular changes in plaque [107]. A similar way to study the characteristics of plaque is through the combination of PET imaging and gene-expression analysis [108]. Future studies will require consistent image–tissue matching, analysis of matched tissue regions, standardised data collection and external validation before any incremental value for patient-level risk stratification or treatment guidance can be established.

## 6. MALDI in Therapeutic Development and Treatment Monitoring

The evidence in this area spans demonstrated preclinical applications, analytical proof-of-concept studies and proposed future clinical applications, which should be interpreted separately.

### 6.1. Finding Targets and Tracking Treatments

MALDI-based lipidomic, metabolomic and proteomic profiling can identify region-specific molecular patterns relevant to therapeutic target discovery. In human and experimental plaques, region-specific differences have been reported in the relative signal intensities and spatial distributions of phosphatidylcholines, lysophospholipids, sphingomyelins and cholesteryl esters. These patterns may help identify pathways of potential therapeutic interest related to lipid uptake, oxidation, breakdown and storage [11,70]. Through the imaging of oxidised phospholipids, it is possible to identify the enzymes that generate or remove damaging lipid products [109]. MALDI-TOF proteomics was used to show the changes in the proteins involved in antioxidant defence, muscle contraction and the structure of the extracellular matrix in different parts of the carotid plaques. The same analysis also detected a fibrinogen fragment, consistent with proteolytic activity within the plaque [110]. These findings may help identify inflammatory mediators, proteolytic enzymes and extracellular-matrix regulators as potential therapeutic targets. A proteomic comparison of stable and unstable plaques found that the inhibition of S100A8/A9 promoted the stabilisation of plaque in mice [111]. The non-imaging proteomic and functional findings described above support target discovery but do not demonstrate intraplaque spatial localisation and are therefore interpreted as complementary rather than direct MALDI-MSI evidence.

Administered drugs and their metabolites can be mapped by MALDI-MSI with arterial sections, all while preserving plaque anatomy. This helps to determine whether exposure has extended into the macrophage-rich regions and the necrotic core or it is limited to just the fibrous cap or vessel media [13]. MALDI-MSI showed that a lipid-rich artificial plaque reduced everolimus transport into the underlying arterial wall [112]. However, this proof-of-concept model used a simplified lipid-containing hydrogel in ex vivo porcine coronary arteries and does not reproduce the full cellular and structural heterogeneity of human atheroma. Its findings should therefore be interpreted primarily as mechanistic evidence of plaque-dependent drug transport. The visualisation of an atherosclerosis-targeted imaging agent within murine plaques has also been possible through MSI [106]. Without the need for radiolabelling, spatial-pharmacological studies have shown that MSI can link the spatial distribution and relative signal intensity of drugs to metabolites and pharmacodynamic responses [113,114].

In experimental and preclinical studies, treatment-related effects can be assessed by comparing MALDI spectra between treated and untreated plaque samples or experimental groups. Isotope-labelled cholesterol imaging has shown esterification and cholesterol uptake that is plaque-specific. This can become a strategy to test lipid-lowering or cholesterol-handling interventions [115]. MALDI-MSI showed that plaque-targeted liposomal docosahexaenoic acid had partly changed the plaque lipid pattern which resembled an earlier stage of the disease. A reduction in the infiltration of macrophages and buildup of lipids in the plaques was also observed [116]. These findings nevertheless remain preclinical and demonstrate local molecular and histological responses rather than established therapeutic efficacy in humans. Similar methods help to assess anti-inflammatory drugs by measuring the changes in oxidised lipids and metabolites related to inflammation. By tracking fibrinogen fragments, platelet-related proteins, lipid signals and other pathway-specific molecular changes, they can also evaluate antithrombotic, metabolic and immune-targeted treatments. Quantitative standards and strong validation against histology, plasma biomarkers and imaging are crucial for the clinical implementation of MALDI.

### 6.2. Targeted Delivery and Precision Treatment

**Figure 3:**
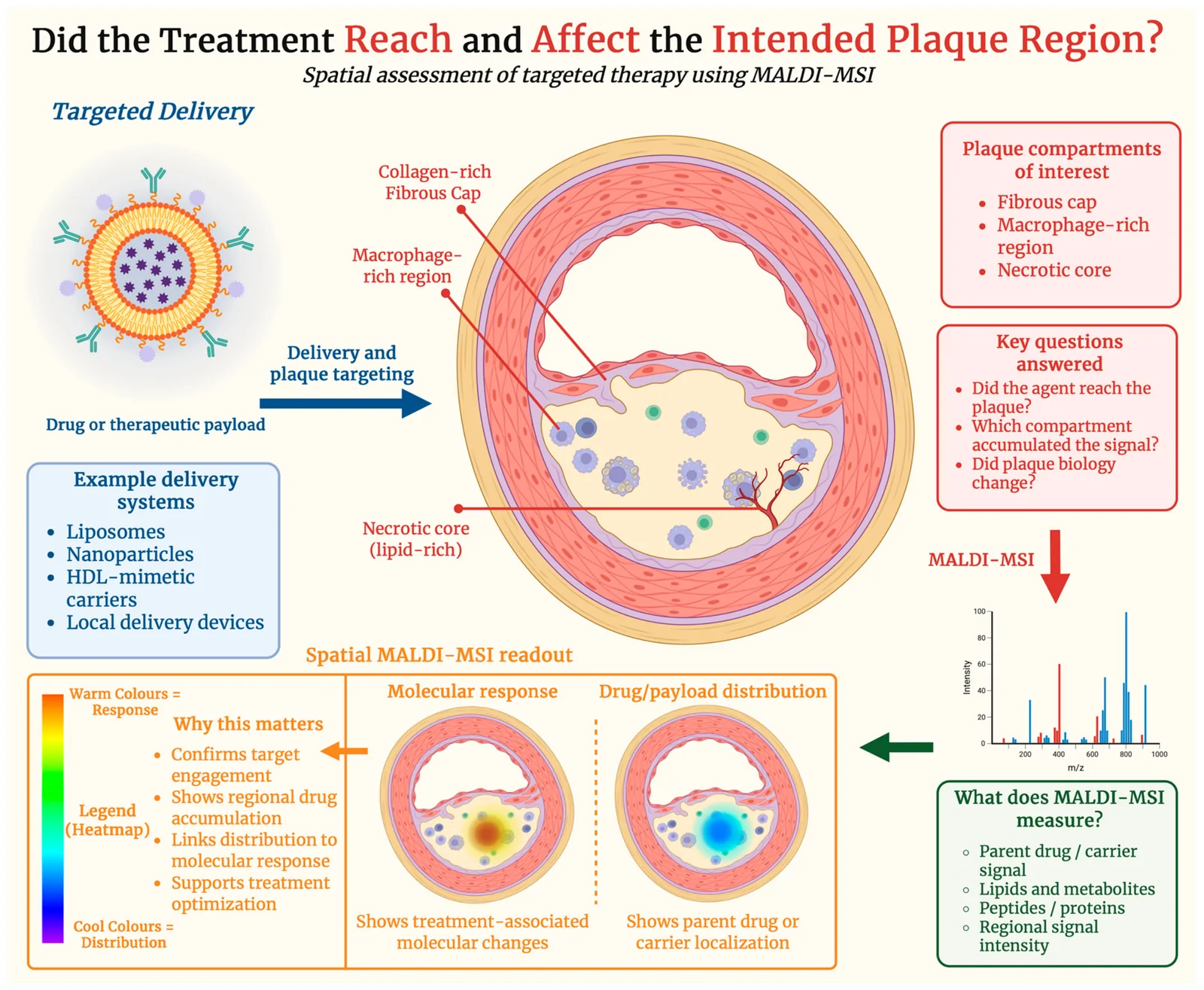
Drug or carrier accumulation can be mapped within specific plaque regions and linked to molecular changes induced by local treatment. Created in BioRender. Roshan, D. (2026) https://BioRender.com/6k6gqxh (accessed on 25 August 2026).

MALDI-MSI can support the preclinical evaluation of plaque-targeted nanoparticles by mapping the spatial distribution of drugs, carrier markers and metabolites. It can be observed whether the drug, nanoparticle marker or metabolite reaches the arterial compartment and builds up in regions rich in macrophages or lipids. Unlike tissue homogenisation, it can differentiate between a parent drug and its metabolites while also preserving its location [117]. Different formulations can be compared using MALDI-MSI by assessing the relative spatial distribution of a drug across different plaque regions. It can also map signals from carriers and changes caused by treatment in lipids or metabolites in areas rich in macrophages, fibrous caps, necrotic cores and calcified regions. Several nanoparticle studies discussed here did not use MALDI-MSI and are included as examples of delivery systems that could subsequently be evaluated spatially. HDL-mimetic PLGA nanoparticles accumulated in plaque macrophages [118], while targeted IL-10 and siRNA nanoparticles produced therapeutic effects in mouse models [119,120]. These studies therefore provide therapeutic context rather than direct evidence of MALDI-MSI-based payload localisation or efficacy. Drugs and degradation products have been mapped by MALDI imaging on drug-eluting stents and explanted porcine arteries in local devices [121]. However, porcine vascular healing occurs considerably faster than human vascular healing, and experimental porcine arteries may not reproduce the advanced atherosclerotic and restenotic pathology encountered clinically. The direct extrapolation of these distribution patterns to human stent responses therefore requires caution [122]. In experimental, ex vivo and device-testing settings, analysis across multiple time points or serial tissue sections can help identify uneven coating, limited penetration, premature loss or prolonged arterial retention from stents, balloons, gels or biodegradable depots. As depicted in Figure 3, MALDI-MSI can be used to determine if a given treatment has reached its intended plaque region and whether its local accumulation has caused any changes in plaque biology.

As a future clinical per spective, MALDI maps could potentially be integrated with clinical imaging and molecular-omics data to support treatment stratification. Human plaques contain distinct molecular and cellular profiles associated with different disease phenotypes [123], while spatial transcriptomic and single-cell studies have identified region-specific pathways and cellular states [124,125]. These latter approaches represent orthogonal non-MALDI evidence and are included to illustrate the potential for multimodal integration with MALDI-MSI rather than to demonstrate that MALDI-MSI currently guides treatment selection. A critical distinction must nevertheless be made between demonstrating spatial target engagement and demonstrating therapeutic efficacy. MALDI-MSI can establish whether a drug or carrier reaches a desired plaque compartment and whether local molecular changes occur, but drug accumulation or pathway modulation alone does not demonstrate plaque stabilisation, improved treatment response or reduced cardiovascular events. Its most established role in this setting therefore remains as a spatial pharmacology and therapeutic-development platform, whereas its ability to guide treatment selection in individual patients remains unproven. The main applications, potential value and current limitations of MALDI in therapeutic development are summarised in Table 4.

## 7. Challenges, Future Perspectives and Conclusions

### 7.1. Current Challenges and Future Directions

MALDI-MSI is vulnerable to pre-analytical and analytical variability that can affect both quantitative measurements and biological interpretation. Ion suppression, matrix composition and deposition conditions influence lipid detection and apparent spatial contrast [126,127]. In human carotid plaques, Greco et al. [11] detected considerably more lipid ions using DHB than norharmane (509 versus 157). Notably, a feature at *m*/*z* 808.58, tentatively assigned to PC(36:2)/PE(39:2), showed opposite associations with symptomatic status in matrix-specific analyses of intimal smooth-muscle-cell regions. Although this discrepancy cannot be attributed exclusively to matrix effects, it illustrates how methodological variability may lead to conflicting interpretations of plaque-associated lipid profiles. Differences in tissue preparation and matrix deposition may additionally influence lipid recovery, ionisation efficiency and spatial localisation [54,59]. Consequently, apparent differences between plaque regions may reflect methodological effects rather than biological variation alone [126,127]. Although absolute quantification remains challenging, automated matrix spraying combined with uniformly applied isotope-labelled internal standards can improve deposition reproducibility and pixel-wise lipid normalisation, with recent workflows incorporating LC–MS/MS quality control [128,129]. On-tissue chemical derivatization offers a complementary strategy for improving the detection and potentially the calibrated quantification of poorly ionising lipids, including cholesterol and fatty acids [130,131]. However, these approaches require further validation in atherosclerotic plaques, where heterogeneous ion suppression, variable derivatization efficiency and potential analyte delocalisation may affect quantitative accuracy. Standardised preparation, appropriate calibration and orthogonal molecular validation therefore remain necessary before quantitative lipid signatures can be reliably linked to plaque vulnerability.

Molecular identification represents a second important limitation. Accurate *m*/*z* matching alone may be insufficient to distinguish isobaric or isomeric lipids and metabolites, particularly when direct on-tissue fragmentation has limited sensitivity. Database-derived assignments should therefore be distinguished from molecular identities confirmed by tandem MS, ion mobility, chemical derivatization or orthogonal LC-MS/MS [132]. This distinction is important when MALDI features are subsequently used to infer biological pathways or proposed as candidate biomarkers, as uncertainty in the original molecular assignment can propagate into the biological interpretation.

Data-processing decisions can also materially affect the molecular patterns obtained from MALDI-MSI. Spectral alignment, baseline correction, normalisation, peak picking, segmentation and the definition of regions of interest influence which features are considered discriminatory [92]. Histological co-registration is therefore essential before signals are attributed to specific plaque compartments or cell populations. Furthermore, increasing spatial resolution requires smaller sampling areas and consequently reduces the amount of analyte available within each pixel, creating a trade-off between spatial detail and sensitivity [133]. These considerations support a hierarchy of evidence in which a detected *m*/*z* feature, a putatively annotated molecule, a histologically co-localised molecular signal and an independently validated biomarker should not be treated as equivalent levels of evidence.

Plaque composition and cellular states vary between regions, patients and arterial beds, complicating the development of universal molecular signatures [134]. Experimental animal models are valuable for mechanistic studies, but their lesions do not fully reproduce the prolonged disease course, comorbidities, rupture, thrombosis or overall complexity of human plaques [135]. Findings from murine or porcine models should therefore be regarded as preclinical evidence until confirmed in human plaque tissue or clinically relevant cohorts. The specialised nature of the field has also resulted in a substantial proportion of the MALDI-MSI literature originating from a relatively small number of research groups, which may limit methodological diversity and the extent of independent replication across laboratories. Many human studies additionally rely on small, cross-sectional collections of surgically excised advanced plaques, making it difficult to determine whether a molecular pattern preceded or resulted from plaque destabilisation. Carotid studies have associated features of excised plaques with subsequent cardiovascular events [136], whereas studies using iliofemoral tissue have produced less consistent findings [137].

Clinical translation would require a standardisation of methods for tissue collection, freezing or fixation, sectioning, washing, matrix application, instrument calibration and storage. As outlined in Section 3.1, this standardisation should remain analyte-specific, because native lipid/metabolite imaging and peptide/protein or glycan imaging require different tissue-processing, washing and matrix-application workflows. Studies carried out in different laboratories have found that the consistency of results and the level of clarity at which small tissue regions can be seen are influenced by tissue digestion and matrix application methods [138]. Reporting frameworks such as minimum information about a mass spectrometry imaging experiment (MIAMSIE)/MSIcheck [139] and SMART [140] should properly document acquisition, processing, normalisation and image-generation parameters. Proposed molecular signatures should first be independently replicated, ideally using blinded validation. They should subsequently be evaluated in large multicentre cohorts for associations with prospective cardiovascular outcomes, including myocardial infarction, stroke, restenosis and cardiovascular death.

In the future, MALDI-2 could increase ionisation efficiency and help to preserve sensitivity during near-single-cell imaging [133]. Integration with spatial transcriptomics and other spatial-omics approaches represents an important next step because these methods provide complementary levels of biological information. Spatial transcriptomics can define region-specific gene-expression programmes and cellular states within atherosclerotic plaques, whereas MALDI-MSI directly maps downstream molecular phenotypes, including lipids, metabolites, peptides and selected proteins. Co-registration of these datasets could therefore link transcriptionally defined macrophage, endothelial and smooth-muscle-cell states with the molecular signatures detected in vulnerable plaque regions [141]. However, sequential MALDI-MSI and spatial transcriptomic analysis on the same section presents practical challenges, as matrix application, laser exposure and subsequent staining may compromise tissue morphology and RNA recovery. Recent same-section workflows have preserved cell identification despite approximately 30% fewer detected transcripts per cell [142]. Serial-section approaches avoid direct assay interference and permit three-dimensional reconstruction [143], but tissue deformation and differences in cellular composition between adjacent sections introduce spatial offsets that computational registration cannot fully eliminate [141]. Consequently, serial-section integration is more appropriate for plaque-compartment or neighbourhood-level comparisons than definitive cellular or subcellular co-localisation. Accurate cell-level integration requires validated same-section protocols, assessment of RNA preservation and registration accuracy, and confirmation of their applicability to atherosclerotic tissue. Multivariate and supervised-learning approaches could support the development of composite plaque vulnerability signatures. However, MALDI-MSI pixels are nested within patients and cannot be treated as independent observations. Mixed-effects models or spatially aware machine-learning approaches should account for intra-patient correlation and spatial dependence, with model training, testing and external validation performed at the patient rather than pixel level.

Accordingly, this review complements the lipid-centred synthesis of Bookmeyer et al. by extending the analysis to proteins, peptides and glycans, biomarker and therapeutic applications, multimodal spatial integration and the evidence required for clinical translation [15]. Within the broader spatial-biology landscape, MALDI-MSI offers broad, label-free molecular coverage but is not universally superior to alternative imaging platforms. DESI-MSI provides similarly label-free lipid and metabolite imaging with minimal sample preparation and can complement or substitute for MALDI in spatial lipidomics. In human atherosclerotic plaques, direct comparison showed broadly complementary performance, with DESI-MSI providing stronger signals for several lipid classes while MALDI-MSI detected some ceramide-related species more effectively [144,145]. TOF-SIMS offers substantially higher spatial resolution and can resolve fine chemical structures within plaques, although greater fragmentation and more limited intact-molecule identification reduce its suitability for broad molecular profiling [144,146].

Imaging mass cytometry provides highly multiplexed, antibody-based protein mapping at cellular resolution, while Visium and MERFISH provide complementary spatial gene-expression and cell-state information [147,148,149]. These approaches can help assign MALDI-derived signatures to specific cellular or transcriptional states, whereas MALDI-MSI directly maps downstream molecular phenotypes. Multimodal integration may therefore provide a more complete interpretation of plaque biology than any single platform alone. The principal strengths and complementary roles of these spatial analytical platforms relative to MALDI-MSI are summarised in Table 5.

### 7.2. Conclusion: From Plaque Mapping to Clinical Care

The central conclusion of this review is that MALDI-MSI is currently more mature as a spatial discovery and pharmacology platform than as a clinical diagnostic or prognostic technology in atherosclerosis. Its principal strength is the ability to resolve molecular heterogeneity that is lost during bulk analysis and to relate lipids, metabolites, peptides, proteins and glycans to defined plaque compartments. Current evidence supports its value for investigating plaque biology, identifying candidate vulnerability and biomarker signatures, and determining the spatial distribution and molecular effects of experimental therapies. However, evidence becomes progressively weaker as these findings move towards individual patient prediction and treatment selection. Small and predominantly cross-sectional cohorts, methodological heterogeneity, incomplete molecular identification and limited independent or prospective validation currently prevent MALDI-derived signatures from being considered clinically actionable. A practical roadmap should proceed in two stages. Over the next 2–3 years, multicentre methodological studies should establish reproducible plaque-MALDI workflows and a minimum reporting dataset including tissue source and plaque phenotype, specimen processing, matrix and deposition method, instrument and calibration parameters, spatial resolution, data preprocessing and normalisation, molecular-identification confidence and histological co-registration. Shared protocols, reference materials and data repositories coordinated through multi-institutional consortia would facilitate inter-laboratory comparison. The subsequent 3–5 years should prioritise prospective multicentre validation of the most reproducible molecular signatures in adequately powered patient cohorts, using predefined cardiovascular endpoints and comparison with established clinical and imaging predictors. Only signatures that demonstrate independent reproducibility and incremental predictive or treatment-response value should progress towards clinical implementation.

## Figures and Tables

**Table 1:** Common matrices used for MALDI-MSI analysis of atherosclerotic tissue.

Matrix	Main MALDI-MSI Application	Typical Polarity	Main Advantages	Important Limitations/Implications
2,5-Dihydroxybenzoic acid (DHB)	Plaque lipids, including phospholipids, sphingolipids and neutral lipids; also applicable to some peptides/glycans	Mainly positive for plaque lipid MSI	Broad lipid coverage; extensively used in cardiovascular MSI; superior coverage to norharmane in direct human carotid-plaque comparison	Matrix/adduct-dependent ionisation; DHB can form relatively large crystals unless deposition is optimised, potentially limiting spatial resolution
α-Cyano-4-hydroxycinnamic acid (CHCA)	Tryptic peptides/proteomic imaging; released N-glycans; selected low-molecular-weight analytes	Mainly positive	Efficient ionisation of peptides and released glycans; fine coatings can support high-resolution imaging	Matrix-related signals may interfere at low *m*/*z*; less comprehensive than DHB for broad plaque lipid profiling
9-Aminoacridine (9-AA)	Acidic phospholipids, fatty acids and metabolites	Mainly negative	Efficient generation of deprotonated ions; useful complement to positive-mode lipid imaging	Limited detection of neutral lipid classes; matrix choice can favour some acidic lipid classes over others
Norharmane	Lipids and glycolipids; complementary lipid profiling	Positive or negative depending on workflow	Broad polarity compatibility; useful as an orthogonal matrix for assessing ionisation bias	In human carotid plaque, yielded fewer lipid peaks than DHB; performance varies with analyte class and deposition conditions

**Table 2:** MALDI technologies that are used in the research of atherosclerosis.

Workflow Level	Representative Approach	Main Role	Samples/Targets	Main Consideration
Instrumentation	MALDI-TOF and MALDI-TOF/TOF	Mass analysis and, with TOF/TOF, fragment-based identification [48]	Tissue extracts, plasma, serum, lipoproteins	Tandem MS can improve molecular identification
Spatial imaging	MALDI-MSI	Acquires spectra across tissue coordinates to reconstruct molecular maps [49]	Arterial tissue; lipids, metabolites, peptides, proteins, glycans	Preserves spatial information
Sample preparation	Fresh-frozen/FFPE processing, washing, matrix application and on-tissue digestion	Prepares analytes for MALDI analysis [58]	Fresh-frozen and paraffin-embedded tissue	Must minimise analyte loss and delocalisation
Molecular identification and data analysis	Accurate mass, databases, MS/MS and LC-MS/MS	Supports molecular annotation and validation [63]	Molecular spectra from plaque tissue	Identification confidence varies; LC-MS/MS offers deeper coverage
Advanced imaging/sensitivity	High-resolution MALDI and MALDI-2	Improves spatial resolution or ionisation efficiency [67]	Lipids, metabolites and low-abundance molecules	Resolution, sensitivity and acquisition-time trade-offs
Targeted/3D imaging extensions	MALDI immunohistochemistry and 3D MALDI-MSI	Targeted molecular localisation and volumetric mapping [69]	Selected proteins and serial tissue sections	Requires accurate spatial/histological alignment

**Table 3:** Representative molecular signatures identified or mapped by MALDI-based approaches in atherosclerosis. Detailed study-level evidence is provided in Supplementary Table S2.

Molecular Category	Representative Molecules or Signals	Main Plaque Region	Reported Pattern	Biological Relevance	Analytical Modality/Study Basis/Identification Evidence
Neutral lipids	Cholesteryl esters and diacylglycerols	Lipid-rich and thrombotic regions	Cholesteryl esters were different between early and advanced plaques, while diacylglycerols increased in thrombotic areas	Indicates lipid storage, plaque progression and thrombosis.	Human carotid tissue, 3 specimens (2 endarterectomy plaques and 1 autopsy sample); lipid identities supported by high-resolution MS and MS/MS [92].
Phospholipids and storage lipids	Phosphatidylcholines, sphingomyelins, lysophosphatidylcholines, cholesteryl esters and triglycerides	Macrophages and cap smooth-muscle cells	Symptomatic plaques contained more phosphatidylcholines and asymptomatic plaques showed more storage lipids	Region-specific lipid differences associated with symptomatic versus asymptomatic clinical status.	Human carotid plaques, n = 29 (9 symptomatic, 20 asymptomatic); symptomatic status defined by TIA symptoms <6 months before surgery; MALDI-MSI with histological and immunofluorescence segmentation [4].
Metabolites and oxidative-stress products	Homoarginine and 2-nonenal	Fibrous cap and necrotic core	Signals annotated as homoarginine were enriched in stable plaques, whereas 2-nonenal-associated signals were enriched in unstable plaques.	Consistent with differences in nitric-oxide-related metabolism and lipid peroxidation.	Human plaques, n = 9 (5 stable, 4 unstable); late-stage type V atheromas classified by fibrous-cap thickness (>200 μm stable; <200 μm unstable); 170 metabolites annotated by database-assisted MSI, with 12 additionally compared with authentic standards by MS/MS [5].
Calcification-associated signatures	Triglycerides, phosphatidic acids, sphingomyelins and thymosin β4	Intima, media and calcified regions	Distinct lipid and protein signals separated arterial layers and calcified areas	Shows molecular changes linked to calcification and remodelling.	Rabbit aortic model, n = 12; molecular assignments based on FT-ICR accurate mass and database/literature matching and reported as tentative where appropriate [61,93]
Proteins and proteolytic products	SOD3, glutathione S-transferase, HSP27 and HSP20	Stable and unstable plaques	Protective antioxidant and stress-response proteins were lower in unstable plaques	Suggests reduced protection against oxidative stress.	Human carotid plaques, n = 48 (19 stable, 29 unstable); stable plaques had a thick fibrous/fibrocalcific cap, whereas unstable plaques had a thin or fissured cap and/or haemorrhagic, ulcerated or thrombotic features; non-imaging 2D electrophoresis followed by MALDI-MS/database identification [94]

**Table 4:** The applications of MALDI in therapeutic development and treatment monitoring.

Therapeutic Application	What MALDI Can Measure	Evidence Type/Experimental Basis	Value for Therapeutic Development	Main Limitation
Identification of therapeutic targets	Lipids, metabolites, proteins and proteolytic fragments	Mixed evidence: direct plaque MALDI-MSI [11,70], non-imaging MALDI proteomics [94,110], and orthogonal LC–MS/MS proteomics [111].	Supports identification of altered inflammatory, lipid and matrix pathways	Candidate targets require functional validation
Spatial assessment of drug distribution	Parent drugs, metabolites and spatial distribution/relative signal intensity	Direct MALDI-MSI proof-of-concept in experimental/ex vivo arterial models [112]	Shows whether drugs reach intended plaque regions	Simplified models and ion suppression limit translation and quantification
Monitoring treatment-induced changes	Changes in lipids, metabolites and response markers	Direct preclinical MALDI-MSI in treated versus untreated experimental tissue [116]	Detects local molecular responses to treatment	Molecular changes do not establish clinical benefit
Plaque-targeted nanoparticles and liposomes	Payloads, carrier signals and lipid changes	Mixed evidence: direct MALDI-MSI for selected formulations; broader nanoparticle studies provide non-MALDI mechanistic context [116,118,119,120]	Supports spatial assessment of delivery and formulation effects	Human therapeutic efficacy has not been established
Local drug-delivery devices	Drug distribution, retention and degradation products	Direct MALDI-MSI proof-of-concept in drug-eluting devices and porcine arterial tissue [121]	Detects distribution and retention of locally delivered drugs	Porcine healing may not reproduce human disease
Exploratory treatment stratification	Combined molecular profiles	Future perspective supported partly by orthogonal transcriptomic and single-cell evidence [123,124,125]	Provides biological rationale for future MALDI-based multimodal stratification	MALDI-guided patient treatment selection and clinical utility remain unvalidated

**Table 5:** Complementary spatial analytical platforms relative to MALDI-MSI.

Platform	Key Strength	Main Limitation	Role Relative to MALDI-MSI
DESI-MSI	Label-free lipid/metabolite imaging with minimal sample preparation	Coverage depends on ionisation and solvent conditions	Can substitute for or validate MALDI spatial lipidomics [144,145]
TOF-SIMS	Very high spatial resolution	Greater fragmentation and more difficult intact-molecule identification	Extends MALDI to finer cellular/subcellular chemical mapping [144,146]
Imaging mass cytometry	Multiplexed protein mapping at cellular resolution	Targeted and antibody-dependent	Validates cellular/protein phenotypes underlying MALDI regions [147]
Spatial transcriptomics (Visium/MERFISH)	Spatial gene-expression and cell-state information	Measures RNA rather than downstream chemical phenotype	Links MALDI molecular signatures to transcriptional programmes and cell states [148,149]

## Data Availability

No new data were created or analysed in this study. Data sharing is not applicable to this article.

## Supplementary Materials

The following supporting information can be downloaded at: https://www.mdpi.com/article/10.3390/ijms27198745/s1.
