# Supplementary material for: Molecular Mapping of Atherosclerotic Plaques with MALDI Mass Spectrometry Imaging
Source: Int J Mol Sci. 2026 Sep 30;27(19):8745. doi: 10.3390/ijms27198745 (PMC13665250; doi:10.3390/ijms27198745)
Supplement: Supplementary file 1 [file ijms-27-08745-s001.zip › ijms-4505779-supplementary.pdf]

Supplementary Table S1. Database Search Strategy

Database	Search strategy
**PubMed – Search 1**	**Query:** atherosclerosis MALDI **Search Details:** ("atherosclerosis"[MeSH Terms] OR "atherosclerosis"[All Fields] OR "atheroscleroses"[All Fields]) AND ("spectrometry, mass, matrix assisted laser desorption ionization"[MeSH Terms] OR ("spectrometry"[All Fields] AND "mass"[All Fields] AND "matrix assisted"[All Fields] AND "laser"[All Fields] AND "desorption ionization"[All Fields]) OR "matrix-assisted laser desorption-ionization mass spectrometry"[All Fields] OR "maldi"[All Fields]) **Filters:** None
**PubMed – Search 2**	**Query:** atherosclerotic plaque MALDI **Search Details:** ("plaque, atherosclerotic"[MeSH Terms] OR ("plaque"[All Fields] AND "atherosclerotic"[All Fields]) OR "atherosclerotic plaque"[All Fields] OR ("atherosclerotic"[All Fields] AND "plaque"[All Fields])) AND ("spectrometry, mass, matrix assisted laser desorption ionization"[MeSH Terms] OR ("spectrometry"[All Fields] AND "mass"[All Fields] AND "matrix assisted"[All Fields] AND "laser"[All Fields] AND "desorption ionization"[All Fields]) OR "matrix-assisted laser desorption-ionization mass spectrometry"[All Fields] OR "maldi"[All Fields]) **Filters:** None
**PubMed – Search 3**	**Query:** atherosclerosis MALDI MSI **Search Details:** ("atherosclerosis"[MeSH Terms] OR "atherosclerosis"[All Fields] OR "atheroscleroses"[All Fields]) AND ("spectrometry, mass, matrix assisted laser desorption ionization"[MeSH Terms] OR ("spectrometry"[All Fields] AND "mass"[All Fields] AND "matrix assisted"[All Fields] AND "laser"[All Fields] AND "desorption ionization"[All Fields]) OR "matrix-assisted laser desorption-ionization mass spectrometry"[All Fields] OR "maldi"[All Fields]) AND "MSI"[All Fields] **Filters:** None
**PubMed – Search 4**	**Query:** atherosclerosis mass spectrometry imaging **Search Details:** ("atherosclerosis"[MeSH Terms] OR "atherosclerosis"[All Fields] OR "atheroscleroses"[All Fields]) AND ("mass spectrometry"[MeSH Terms] OR ("mass"[All Fields] AND "spectrometry"[All Fields]) OR "mass spectrometry"[All Fields]) AND ("image s"[All Fields] OR "imaged"[All Fields] OR "imager"[All Fields] OR "imager s"[All Fields] OR "imagers"[All Fields] OR "images"[All Fields] OR "imaging"[All Fields] OR "imaging s"[All Fields] OR "imagings"[All Fields] OR "intrauterine growth retardation metaphyseal dysplasia adrenal hypoplasia congenita and genital anomalies"[Supplementary Concept] OR "intrauterine growth retardation metaphyseal dysplasia adrenal hypoplasia congenita and genital anomalies"[All Fields] OR "image"[All Fields]) **Filters:** None
**PubMed – Search 5**	**Query:** atherosclerotic plaque mass spectrometry imaging **Search Details:** ("plaque, atherosclerotic"[MeSH Terms] OR ("plaque"[All Fields] AND "atherosclerotic"[All Fields]) OR "atherosclerotic plaque"[All Fields] OR ("atherosclerotic"[All Fields] AND "plaque"[All Fields])) AND ("mass spectrometry"[MeSH Terms] OR ("mass"[All Fields] AND "spectrometry"[All Fields]) OR "mass spectrometry"[All Fields]) AND ("image s"[All Fields] OR "imaged"[All Fields] OR "imager"[All Fields] OR "imager s"[All Fields] OR "imagers"[All Fields] OR "images"[All Fields] OR "imaging"[All Fields] OR "imaging s"[All Fields] OR "imagings"[All Fields] OR "intrauterine growth retardation metaphyseal dysplasia adrenal hypoplasia congenita and genital anomalies"[Supplementary Concept] OR "intrauterine growth retardation metaphyseal dysplasia adrenal hypoplasia congenita and genital anomalies"[All Fields] OR "image"[All Fields]) **Filters:** None

**PubMed – Search 6**	**Query:** carotid plaque MALDI **Search Details:** ("carotid arteries"[MeSH Terms] OR ("carotid"[All Fields] AND "arteries"[All Fields]) OR "carotid arteries"[All Fields] OR "carotid"[All Fields] OR "carotids"[All Fields] OR "carotidal"[All Fields] OR "carotide"[All Fields] OR "carotideal"[All Fields]) AND ("plaque s"[All Fields] OR "plaque, amyloid"[MeSH Terms] OR ("plaque"[All Fields] AND "amyloid"[All Fields]) OR "amyloid plaque"[All Fields] OR "plaque"[All Fields] OR "dental plaque"[MeSH Terms] OR ("dental"[All Fields] AND "plaque"[All Fields]) OR "dental plaque"[All Fields] OR "plaques"[All Fields]) AND ("spectrometry, mass, matrix assisted laser desorption ionization"[MeSH Terms] OR ("spectrometry"[All Fields] AND "mass"[All Fields] AND "matrix assisted"[All Fields] AND "laser"[All Fields] AND "desorption ionization"[All Fields]) OR "matrix-assisted laser desorption-ionization mass spectrometry"[All Fields] OR "maldi"[All Fields]) **Filters:** None
**PubMed – Search 7**	**Query:** carotid plaque mass spectrometry imaging **Search Details:** ("carotid arteries"[MeSH Terms] OR ("carotid"[All Fields] AND "arteries"[All Fields]) OR "carotid arteries"[All Fields] OR "carotid"[All Fields] OR "carotids"[All Fields] OR "carotidal"[All Fields] OR "carotide"[All Fields] OR "carotideal"[All Fields]) AND ("plaque s"[All Fields] OR "plaque, amyloid"[MeSH Terms] OR ("plaque"[All Fields] AND "amyloid"[All Fields]) OR "amyloid plaque"[All Fields] OR "plaque"[All Fields] OR "dental plaque"[MeSH Terms] OR ("dental"[All Fields] AND "plaque"[All Fields]) OR "dental plaque"[All Fields] OR "plaques"[All Fields]) AND ("mass spectrometry"[MeSH Terms] OR ("mass"[All Fields] AND "spectrometry"[All Fields]) OR "mass spectrometry"[All Fields]) AND ("image s"[All Fields] OR "imaged"[All Fields] OR "imager"[All Fields] OR "imager s"[All Fields] OR "imagers"[All Fields] OR "images"[All Fields] OR "imaging"[All Fields] OR "imaging s"[All Fields] OR "imagings"[All Fields] OR "intrauterine growth retardation metaphyseal dysplasia adrenal hypoplasia congenita and genital anomalies"[Supplementary Concept] OR "intrauterine growth retardation metaphyseal dysplasia adrenal hypoplasia congenita and genital anomalies"[All Fields] OR "image"[All Fields]) **Filters:** None
**PubMed – Search 8**	**Query:** coronary plaque MALDI **Search Details:** ("coronaries"[All Fields] OR "heart"[MeSH Terms] OR "heart"[All Fields] OR "coronary"[All Fields]) AND ("plaque s"[All Fields] OR "plaque, amyloid"[MeSH Terms] OR ("plaque"[All Fields] AND "amyloid"[All Fields]) OR "amyloid plaque"[All Fields] OR "plaque"[All Fields] OR "dental plaque"[MeSH Terms] OR ("dental"[All Fields] AND "plaque"[All Fields]) OR "dental plaque"[All Fields] OR "plaques"[All Fields]) AND ("spectrometry, mass, matrix assisted laser desorption ionization"[MeSH Terms] OR ("spectrometry"[All Fields] AND "mass"[All Fields] AND "matrix assisted"[All Fields] AND "laser"[All Fields] AND "desorption ionization"[All Fields]) OR "matrix-assisted laser desorption-ionization mass spectrometry"[All Fields] OR "maldi"[All Fields]) **Filters:** None
**PubMed – Search 9**	**Query:** coronary plaque mass spectrometry imaging **Search Details:** ("coronaries"[All Fields] OR "heart"[MeSH Terms] OR "heart"[All Fields] OR "coronary"[All Fields]) AND ("plaque s"[All Fields] OR "plaque, amyloid"[MeSH Terms] OR ("plaque"[All Fields] AND "amyloid"[All Fields]) OR "amyloid plaque"[All Fields] OR "plaque"[All Fields] OR "dental plaque"[MeSH Terms] OR ("dental"[All Fields] AND "plaque"[All Fields]) OR "dental plaque"[All Fields] OR "plaques"[All Fields]) AND ("mass spectrometry"[MeSH Terms] OR ("mass"[All Fields] AND "spectrometry"[All Fields]) OR "mass spectrometry"[All Fields]) AND ("image s"[All Fields] OR "imaged"[All Fields] OR "imager"[All Fields] OR "imager s"[All Fields] OR "imagers"[All Fields] OR "images"[All Fields] OR "imaging"[All Fields] OR "imaging s"[All Fields] OR "imagings"[All Fields] OR "intrauterine growth retardation metaphyseal dysplasia adrenal hypoplasia congenita

	and genital anomalies"[Supplementary Concept] OR "intrauterine growth retardation metaphyseal dysplasia adrenal hypoplasia congenita and genital anomalies"[All Fields] OR "image"[All Fields]) **Filters:**None
**PubMed – Search 10**	**Query:** atherosclerosis MALDI lipidomics **Search Details:** ("atherosclerosis"[MeSH Terms] OR "atherosclerosis"[All Fields] OR "atheroscleroses"[All Fields]) AND ("spectrometry, mass, matrix assisted laser desorption ionization"[MeSH Terms] OR ("spectrometry"[All Fields] AND "mass"[All Fields] AND "matrix assisted"[All Fields] AND "laser"[All Fields] AND "desorption ionization"[All Fields]) OR "matrix-assisted laser desorption-ionization mass spectrometry"[All Fields] OR "maldi"[All Fields]) AND ("lipidomics"[MeSH Terms] OR "lipidomics"[All Fields] OR "lipidome"[All Fields] OR "lipidomes"[All Fields] OR "lipidomic"[All Fields]) **Filters:** None
**PubMed – Search 11**	**Query:** atherosclerotic plaque MALDI lipidomics **Search Details:** ("plaque, atherosclerotic"[MeSH Terms] OR ("plaque"[All Fields] AND "atherosclerotic"[All Fields]) OR "atherosclerotic plaque"[All Fields] OR ("atherosclerotic"[All Fields] AND "plaque"[All Fields])) AND ("spectrometry, mass, matrix assisted laser desorption ionization"[MeSH Terms] OR ("spectrometry"[All Fields] AND "mass"[All Fields] AND "matrix assisted"[All Fields] AND "laser"[All Fields] AND "desorption ionization"[All Fields]) OR "matrix-assisted laser desorption-ionization mass spectrometry"[All Fields] OR "maldi"[All Fields]) AND ("lipidomics"[MeSH Terms] OR "lipidomics"[All Fields] OR "lipidome"[All Fields] OR "lipidomes"[All Fields] OR "lipidomic"[All Fields]) **Filters:** None
**PubMed – Search 12**	**Query:** atherosclerosis MALDI metabolomics **Search Details:** ("atherosclerosis"[MeSH Terms] OR "atherosclerosis"[All Fields] OR "atheroscleroses"[All Fields]) AND ("spectrometry, mass, matrix assisted laser desorption ionization"[MeSH Terms] OR ("spectrometry"[All Fields] AND "mass"[All Fields] AND "matrix assisted"[All Fields] AND "laser"[All Fields] AND "desorption ionization"[All Fields]) OR "matrix-assisted laser desorption-ionization mass spectrometry"[All Fields] OR "maldi"[All Fields]) AND ("metabolome"[MeSH Terms] OR "metabolome"[All Fields] OR "metabolomes"[All Fields] OR "metabolomics"[MeSH Terms] OR "metabolomics"[All Fields] OR "metabolomic"[All Fields]) **Filters:** None
**PubMed – Search 13**	**Query:** atherosclerotic plaque MALDI metabolomics **Search Details:** ("plaque, atherosclerotic"[MeSH Terms] OR ("plaque"[All Fields] AND "atherosclerotic"[All Fields]) OR "atherosclerotic plaque"[All Fields] OR ("atherosclerotic"[All Fields] AND "plaque"[All Fields])) AND ("spectrometry, mass, matrix assisted laser desorption ionization"[MeSH Terms] OR ("spectrometry"[All Fields] AND "mass"[All Fields] AND "matrix assisted"[All Fields] AND "laser"[All Fields] AND "desorption ionization"[All Fields]) OR "matrix-assisted laser desorption-ionization mass spectrometry"[All Fields] OR "maldi"[All Fields]) AND ("metabolome"[MeSH Terms] OR "metabolome"[All Fields] OR "metabolomes"[All Fields] OR "metabolomics"[MeSH Terms] OR "metabolomics"[All Fields] OR "metabolomic"[All Fields]) **Filters:** None
**PubMed – Search 14**	**Query:** atherosclerosis MALDI proteomics **Search Details:** ("atherosclerosis"[MeSH Terms] OR "atherosclerosis"[All Fields] OR "atheroscleroses"[All Fields]) AND ("spectrometry, mass, matrix assisted laser desorption ionization"[MeSH Terms] OR ("spectrometry"[All Fields] AND "mass"[All Fields] AND "matrix assisted"[All Fields] AND "laser"[All Fields] AND "desorption ionization"[All Fields]) OR "matrix-assisted laser desorption-ionization mass spectrometry"[All Fields] OR "maldi"[All Fields]) AND ("proteom"[All Fields] OR "proteome"[Supplementary Concept] OR "proteome"[All Fields] OR "proteome"[MeSH Terms] OR "proteomes"[All Fields] OR "proteomical"[All Fields] OR "proteomically"[All Fields] OR "proteomics"[MeSH Terms] OR "proteomics"[All Fields] OR "proteomic"[All Fields]) **Filters:** None

**PubMed – Search 15**	**Query:** atherosclerotic plaque MALDI proteomics **Search Details:** ("plaque, atherosclerotic"[MeSH Terms] OR ("plaque"[All Fields] AND "atherosclerotic"[All Fields]) OR "atherosclerotic plaque"[All Fields] OR ("atherosclerotic"[All Fields] AND "plaque"[All Fields])) AND ("spectrometry, mass, matrix assisted laser desorption ionization"[MeSH Terms] OR ("spectrometry"[All Fields] AND "mass"[All Fields] AND "matrix assisted"[All Fields] AND "laser"[All Fields] AND "desorption ionization"[All Fields]) OR "matrix-assisted laser desorption-ionization mass spectrometry"[All Fields] OR "maldi"[All Fields]) AND ("proteom"[All Fields] OR "proteome"[Supplementary Concept] OR "proteome"[All Fields] OR "proteome"[MeSH Terms] OR "proteomes"[All Fields] OR "proteomical"[All Fields] OR "proteomically"[All Fields] OR "proteomics"[MeSH Terms] OR "proteomics"[All Fields] OR "proteomic"[All Fields]) **Filters:** None
**PubMed – Search 16**	**Query:** atherosclerosis MALDI TOF **Search Details:** ("atherosclerosis"[MeSH Terms] OR "atherosclerosis"[All Fields] OR "atheroscleroses"[All Fields]) AND ("spectrometry, mass, matrix assisted laser desorption ionization"[MeSH Terms] OR ("spectrometry"[All Fields] AND "mass"[All Fields] AND "matrix assisted"[All Fields] AND "laser"[All Fields] AND "desorption ionization"[All Fields]) OR "matrix-assisted laser desorption-ionization mass spectrometry"[All Fields] OR "maldi"[All Fields]) AND "TOF"[All Fields] **Filters:** None
**PubMed – Search 17**	**Query:** atherosclerosis MALDI biomarker **Search Details:** ("atherosclerosis"[MeSH Terms] OR "atherosclerosis"[All Fields] OR "atheroscleroses"[All Fields]) AND ("spectrometry, mass, matrix assisted laser desorption ionization"[MeSH Terms] OR ("spectrometry"[All Fields] AND "mass"[All Fields] AND "matrix assisted"[All Fields] AND "laser"[All Fields] AND "desorption ionization"[All Fields]) OR "matrix-assisted laser desorption-ionization mass spectrometry"[All Fields] OR "maldi"[All Fields]) AND ("biomarker s"[All Fields] OR "biomarkers"[Supplementary Concept] OR "biomarkers"[All Fields] OR "biomarker"[All Fields] OR "biomarkers"[MeSH Terms]) **Filters:** None
**PubMed – Search 18**	**Query:** atherosclerosis MALDI plasma **Search Details:** ("atherosclerosis"[MeSH Terms] OR "atherosclerosis"[All Fields] OR "atheroscleroses"[All Fields]) AND ("spectrometry, mass, matrix assisted laser desorption ionization"[MeSH Terms] OR ("spectrometry"[All Fields] AND "mass"[All Fields] AND "matrix assisted"[All Fields] AND "laser"[All Fields] AND "desorption ionization"[All Fields]) OR "matrix-assisted laser desorption-ionization mass spectrometry"[All Fields] OR "maldi"[All Fields]) AND ("plasma"[MeSH Terms] OR "plasma"[All Fields] OR "plasmas"[All Fields] OR "plasma s"[All Fields]) **Filters:** None
**PubMed – Search 19**	**Query:** atherosclerosis MALDI serum **Search Details:** ("atherosclerosis"[MeSH Terms] OR "atherosclerosis"[All Fields] OR "atheroscleroses"[All Fields]) AND ("spectrometry, mass, matrix assisted laser desorption ionization"[MeSH Terms] OR ("spectrometry"[All Fields] AND "mass"[All Fields] AND "matrix assisted"[All Fields] AND "laser"[All Fields] AND "desorption ionization"[All Fields]) OR "matrix-assisted laser desorption-ionization mass spectrometry"[All Fields] OR "maldi"[All Fields]) AND ("serum"[MeSH Terms] OR "serum"[All Fields] OR "serums"[All Fields] OR "serum s"[All Fields] OR "serumal"[All Fields]) **Filters:**None
**PubMed – Search 20**	**Query:** atherosclerosis MALDI lipoprotein **Search Details:** ("atherosclerosis"[MeSH Terms] OR "atherosclerosis"[All Fields] OR "atheroscleroses"[All Fields]) AND ("spectrometry, mass, matrix assisted laser desorption ionization"[MeSH Terms] OR ("spectrometry"[All Fields] AND "mass"[All Fields] AND "matrix assisted"[All Fields] AND "laser"[All Fields] AND "desorption ionization"[All Fields]) OR "matrix-assisted laser desorption-ionization mass spectrometry"[All Fields] OR "maldi"[All Fields]) AND ("lipoprotein s"[All Fields] OR "lipoproteine"[All Fields] OR "lipoproteins"[Supplementary Concept] OR "lipoproteins"[All Fields] OR "lipoprotein"[All Fields] OR "lipoproteins"[MeSH Terms]) **Filters:** None

**PubMed – Search 21**	**Query:** atherosclerosis MALDI drug **Search Details:** ("atherosclerosis"[MeSH Terms] OR "atherosclerosis"[All Fields] OR "atheroscleroses"[All Fields]) AND ("spectrometry, mass, matrix assisted laser desorption ionization"[MeSH Terms] OR ("spectrometry"[All Fields] AND "mass"[All Fields] AND "matrix assisted"[All Fields] AND "laser"[All Fields] AND "desorption ionization"[All Fields]) OR "matrix-assisted laser desorption-ionization mass spectrometry"[All Fields] OR "maldi"[All Fields]) AND "drug"[All Fields] **Filters:** None
**PubMed – Search 22**	**Query:** atherosclerosis mass spectrometry imaging drug **Search Details:** ("atherosclerosis"[MeSH Terms] OR "atherosclerosis"[All Fields] OR "atheroscleroses"[All Fields]) AND ("mass spectrometry"[MeSH Terms] OR ("mass"[All Fields] AND "spectrometry"[All Fields]) OR "mass spectrometry"[All Fields]) AND ("image s"[All Fields] OR "imaged"[All Fields] OR "imager"[All Fields] OR "imager s"[All Fields] OR "imagers"[All Fields] OR "images"[All Fields] OR "imaging"[All Fields] OR "imaging s"[All Fields] OR "imagings"[All Fields] OR "intrauterine growth retardation metaphyseal dysplasia adrenal hypoplasia congenita and genital anomalies"[Supplementary Concept] OR "intrauterine growth retardation metaphyseal dysplasia adrenal hypoplasia congenita and genital anomalies"[All Fields] OR "image"[All Fields]) AND "drug"[All Fields] **Filters:** None

Supplementary Figure S1. Flow Diagram depicting the selection of primary MALDI publications.

PRISMA-style flow diagram illustrating the identification, screening, eligibility assessment and selection of primary MALDI-based publications for the structured narrative review. Literature searches were conducted in PubMed and PubMed Central (PMC) from database inception to 14 September 2026. Following duplicate removal, records underwent title and abstract screening and subsequent full-text assessment according to predefined eligibility criteria. Publications included as primary MALDI evidence were distinguished from those used exclusively for methodological or biological context.

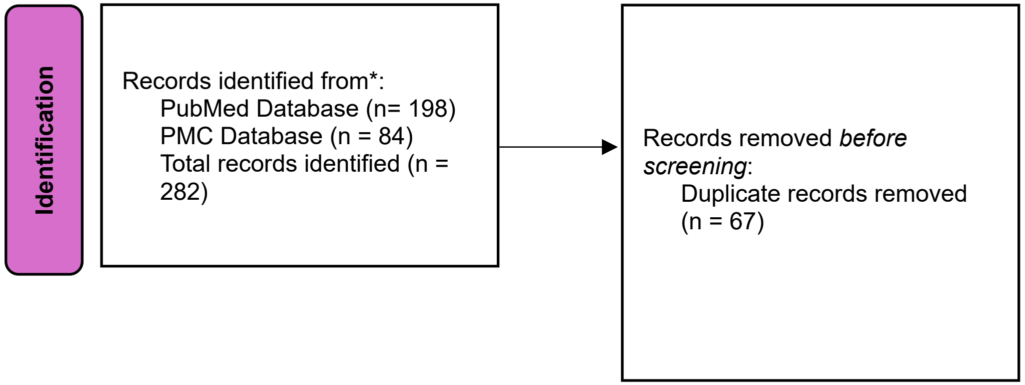

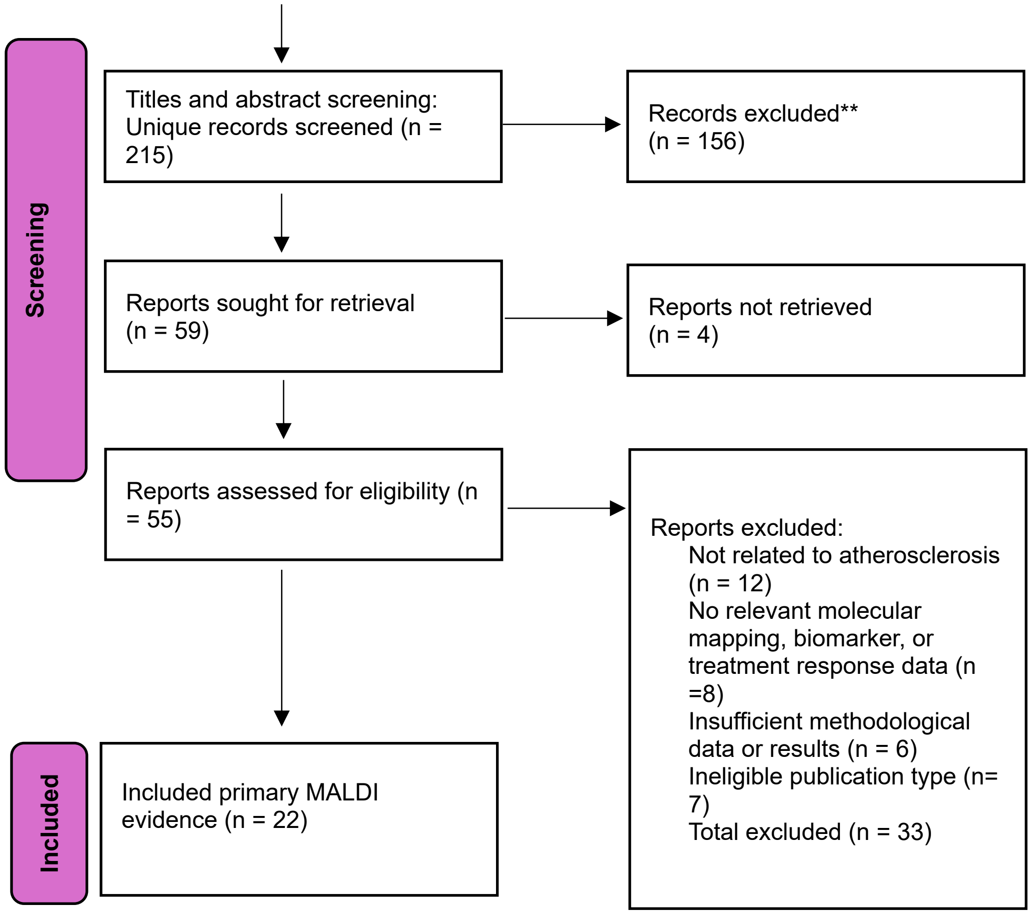
Screening
Titles and abstract screening: Unique records screened (n = 215)
Records excluded** (n = 156)
Reports sought for retrieval (n = 59)
Reports not retrieved (n = 4)
Reports assessed for eligibility (n = 55)
Reports excluded:
Not related to atherosclerosis (n = 12)
No relevant molecular mapping, biomarker, or treatment response data (n =8)
Insufficient methodological data or results (n = 6)
Ineligible publication type (n= 7)
Total excluded (n = 33)
Included
Included primary MALDI evidence (n = 22)

Supplementary Table S2. Study-level characteristics of primary MALDI-based studies relevant to atherosclerosis included in the narrative synthesis.

This table summarises the primary MALDI-based studies contributing directly to the evidence synthesis. References used only for background pathophysiology, methodological context, biological interpretation or complementary non-MALDI evidence are not considered part of the primary MALDI evidence corpus.

A. Human plaque and arterial-tissue spatial MALDI-MSI studies

First author, year [Ref.]	Study/model, species and sample size	Arterial bed / plaque phenotype	MALDI modality and principal analytical characteristics	Analyte class	Molecular identification / validation	Principal findings	Principal limitations
**Greco, 2024 [4]**	Human carotid endarterectomy; n = 29 patients (9 symptomatic, 20 asymptomatic)	Carotid plaques; symptomatic status defined by TIA within 6 months before surgery versus asymptomatic plaques; histologically defined macrophage-rich and VSMC-rich regions	Spatial lipidomics by MALDI-MSI, co-registered with histology and immunofluorescence to segment histomolecular plaque regions	Lipids, including phosphatidylcholines, lysophosphatidylcholines, cholesteryl esters and triglycerides	Study-specific lipid annotation combined with histological/immunofluorescence localisation	Macrophage-rich regions of symptomatic plaques were enriched in phosphatidylcholines, whereas corresponding regions of asymptomatic plaques contained more polyunsaturated cholesteryl esters and triglycerides; fibrous-cap VSMC regions of asymptomatic plaques were enriched in lysophosphatidylcholines and cholesteryl esters	Cross-sectional surgical cohort; symptomatic status is not equivalent to histological instability or prospective rupture risk; unequal group sizes; no independent prospective validation cohort.

First author, year [Ref.]	Study/model, species and sample size	Arterial bed / plaque phenotype	MALDI modality and principal analytical characteristics	Analyte class	Molecular identification / validation	Principal findings	Principal limitations
**Seeley, 2023 [5]**	Human atherosclerotic tissue from n = 9 unmatched individuals; 5 stable and 4 unstable late-stage atheromas	Coronary/carotid arterial biobank specimens; type V lesions; stable defined by fibrous cap >200 μm and unstable by cap <200 μm	MALDI-MSI using timsTOF fleX; positive- and negative-ion acquisition; FFPE tissue; spatially resolved analysis of fibrous cap and necrotic core	Metabolites, including lipids, amino-acid-related metabolites and oxidative-stress-associated products	855 peaks with chemical formula assignments; 170 metabolites annotated using MetaboScape, METASPACE and HMDB; 12 metabolites additionally compared with authentic standards by MS/MS	Stable plaques were enriched in lipid metabolism, long-chain fatty-acid pathways, acylcarnitines and acylglycines; unstable plaques showed greater ROS-, aromatic-amino-acid- and tryptophan-related metabolism; homoarginine-associated signals favoured stable plaques and 2-nonenal-associated signals unstable plaques	Very small cohort; unmatched specimens; FFPE processing may alter molecular coverage; many identifications remain annotation-level rather than authentic-standard-confirmed; no independent validation cohort.
**Greco, 2021 [11]**	Human carotid endarterectomy; n = 6 plaques	Symptomatic and asymptomatic carotid plaques spanning AHA lesion classes; macrophage-rich, VSMC-rich, haemorrhagic, necrotic and collagen-rich regions examined	High-resolution MALDI-MSI of fresh-frozen plaque; DHB and norharmane matrices directly compared; adjacent histology and immunofluorescence used for regional segmentation	Primarily lipids	High-resolution mass-based lipid annotation; complementary matrices used to assess matrix-dependent coverage	Demonstrated marked regional lipid heterogeneity; macrophage-rich and intimal VSMC regions showed prominent plaque-associated lipid differences, supporting region-specific rather than bulk plaque analysis	n = 6 exploratory cohort; cross-sectional excised tissue; matrix-dependent ionisation affects detectable lipid coverage; no external or prospective clinical validation.

First author, year [Ref.]	Study/model, species and sample size	Arterial bed / plaque phenotype	MALDI modality and principal analytical characteristics	Analyte class	Molecular identification / validation	Principal findings	Principal limitations
**Martin-Lorenzo, 2014 [54]**	Human healthy and atherosclerotic arterial tissue; technical and biological repeats were used. The exact number of independent biological samples was not stated in the published abstract; full-text verification was not possible.	Human arterial wall; healthy versus atherosclerotic tissue; intima and media	Protein MALDI-MSI at 30 μm spatial resolution; five tissue-preparation protocols compared in a randomised workflow	Proteins	Protein MSI after systematic sample-preparation optimisation; principal aim was analytical benchmarking rather than biomarker validation	Established an optimised preparation workflow enabling 30 μm protein imaging and demonstrated layer-specific protein signals in atherosclerotic arterial tissue	Primarily a methodological/technical study; limited biological cohort information; no clinical-outcome analysis; not designed to validate disease biomarkers.
**Moerman, 2021 [70]**	Human carotid endarterectomy; n = 12 plaques, represented by 106 tissue sections	Advanced carotid atherosclerosis; necrotic core, foam-cell-rich areas, fibrous tissue, fibrin, erythrocyte-rich and other plaque compartments	MALDI-MSI on Synapt G2Si-TOF; positive ion mode; approximately 45 × 45 μm pixels; histological co-registration; m/z 300–1200	Lipids	194 lipid-related signals detected; >90 uniquely identified or assigned lipid species; high-resolution analytical support used for selected assignments	Revealed extensive spatially organised lipid signatures across advanced plaque compartments, including distinct patterns associated with necrotic, foam-cell-rich and fibrous regions	Only 12 independent patients despite 106 sections; repeated sections are not independent biological replicates; cross-sectional excised tissue; no prospective clinical validation.

First author, year [Ref.]	Study/model, species and sample size	Arterial bed / plaque phenotype	MALDI modality and principal analytical characteristics	Analyte class	Molecular identification / validation	Principal findings	Principal limitations
**Visscher, 2019 [92]**	Human carotid tissue; n = 3 specimens (2 advanced endarterectomy plaques and 1 early-stage autopsy specimen)	Carotid artery; early versus advanced plaque and intraplaque regions including thrombotic areas	MALDI-MSI; systematic tissue-preparation and data-processing pipeline; high-resolution lipid imaging with histological comparison	Lipids	High-resolution MS and MS/MS-supported lipid assignments within a data-processing workflow	Diacylglycerols were more abundant in a thrombotic area and cholesteryl-ester composition distinguished advanced from early-stage plaque; established a reproducible processing pipeline for carotid lipid MSI	Extremely small pilot dataset (n = 3); primarily methodological; no independent validation or prospective outcome testing.
**Slijkhuis, 2025 [140]**	Human carotid endarterectomy; n = 4 plaques	Human carotid atherosclerotic plaque	Head-to-head MALDI-MSI versus DESI-MSI on a SYNAPT XS; positive-ion mode; MALDI used DHB matrix; spectral sensitivity and image quality compared	Lipids	Comparative lipid detection/annotation across two ionisation approaches	Both approaches provided broad plaque-lipid coverage; DESI generally produced higher ion counts and sharper images, whereas MALDI showed comparatively greater detection of some ceramide and hexosylceramide species	**n = 4**; primarily an analytical comparison rather than disease-validation study; detected lipid profile depends on ionisation method; no clinical endpoint assessment.

B. Animal and mixed human–animal spatial MALDI-MSI studies

First author, year [Ref.]	Study/model, species and sample size	Arterial bed / plaque phenotype	MALDI modality and principal analytical characteristics	Analyte class	Molecular identification / validation	Principal findings	Principal limitations
**Patterson, 2016 [12]**	Human carotid-plaque dataset and mouse aortic-sinus dataset; proof-of-concept 3D datasets	Human carotid plaque and murine aortic-sinus atherosclerosis	Three-dimensional MALDI imaging MS reconstructed from serial sections; open-source reconstruction and multivariate segmentation workflow	Lipids	Molecular imaging combined with computational 3D reconstruction; identification was secondary to development of the spatial reconstruction methodology	Demonstrated that plaque-associated lipid distributions can be reconstructed in three dimensions and interrogated across serial tissue sections	Primarily a methodological proof-of-concept; limited biological sample numbers; serial-section alignment and section-to-section variability can affect 3D reconstruction; not designed for clinical phenotype validation.

First author, year [Ref.]	Study/model, species and sample size	Arterial bed / plaque phenotype	MALDI modality and principal analytical characteristics	Analyte class	Molecular identification / validation	Principal findings	Principal limitations
**Khamehgir-Silz, 2022 [52]**	Mouse: old ApoE−/− n = 4, young ApoE−/− n = 2, WT n = 6; human: 8 atherosclerotic and 3 control arterial specimens	Mouse aortic arch/carotid branches; human peripheral arteries from amputated limbs with or without atherosclerotic changes	High-resolution atmospheric-pressure MALDI-MSI/Orbitrap; positive ion mode; mass resolution 140,000–240,000 at m/z 200; ±3 ppm mass accuracy; 7 μm mouse and 5–15 μm human spatial resolution	Cholesteryl esters, lysophosphatidylcholines, lysophosphatidylethanolamines and cholesterol derivatives	Accurate-mass/database assignment; on-tissue MS experiments attempted but marker intensities were insufficient for successful fragmentation, so many assignments remained putative	Thirty-one plaque-associated m/z values were specific to old ApoE−/− mice; 26 were also detected in human diseased vessels; macrophage-rich human regions showed characteristic lipid enrichment	Small animal and human cohorts; human tissue derived from amputated peripheral limbs rather than carotid/coronary plaques; treatment and clinical heterogeneity; species differences; on-tissue MS/MS confirmation was unsuccessful for many proposed markers.

First author, year [Ref.]	Study/model, species and sample size	Arterial bed / plaque phenotype	MALDI modality and principal analytical characteristics	Analyte class	Molecular identification / validation	Principal findings	Principal limitations
**Martin-Lorenzo, 2015 [61,93]**	Rabbit model; **n** = 12 New Zealand White rabbits, control versus cholesterol-enriched diet; 36 sections analysed (3/animal)	Ascending aorta; early experimental atherosclerosis; intima, media and calcified intimal regions	High-spatial-resolution MALDI-MSI (~30 µm) using separate protein, lipid and metabolite protocols; FT-ICR used for high-accuracy measurements	Lipids, metabolites and proteins	FT-ICR accurate mass plus MSiMass, MaTisse, LIPID MAPS and literature matching; assignments explicitly tentative where appropriate; selected protein finding confirmed by immunohistochemistry	Demonstrated distinct molecular signatures of intima, media and calcified regions; identified accumulation of saturated fatty acids, lysolipids and several phospholipid classes and localised increased TMSB4X in atherosclerotic intima	Early rabbit atherosclerosis does not reproduce advanced human plaque complexity or rupture biology; molecular identifications were not uniformly definitive; multiple sections from the same animals are not independent subjects. Refs. [61] and [93] represent companion reports from this same study and should be treated as one evidence unit.

First author, year [Ref.]	Study/model, species and sample size	Arterial bed / plaque phenotype	MALDI modality and principal analytical characteristics	Analyte class	Molecular identification / validation	Principal findings	Principal limitations
**Cao, 2020 [71]**	15 Ldlr−/− and 4 Apoe−/− mice for 2D comparisons; 3D reconstruction additionally demonstrated	Aortic root atheroma; necrotic core and surrounding plaque regions	High-spatial-resolution 2D and 3D MALDI-MSI, positive- and negative-ion modes	Plaque lipids, particularly sphingomyelin and lysophospholipids	High-resolution MS1/MS2 used to identify shared plaque-associated m/z features	Twenty-five plaque-associated m/z species were common to both mouse models; identified lipids included SM, LPA, LPC, LPE and LPI species, with LPI(18:0) preferentially localised to necrotic core	Entirely murine; model genotype and dietary conditions differ; limited generalisability to human disease; 3D demonstration involved much less biological replication than the 2D analysis.
**Slijkhuis, 2024 [72]**	Familial-hypercholesterolaemia swine; n = 6 animals; 17 coronary-artery sections classified as healthy (n = 6), mild (n = 6) or advanced (n = 5)	Coronary arteries spanning healthy, mild and advanced plaque; necrotic-core and inflammatory-cell regions analysed	MALDI-MSI, positive and negative modes; Synapt G2Si-TOF; DHB positive / 1,5-DAN negative; 45 × 45 µm pixels; m/z 300–1200; histology and NMF/OPLS-based analysis	Lipids	473 lipid-related features detected; lipid annotation supported by analytical workflow and higher-resolution measurements for selected tissue	Disease stage showed distinct lipid signatures; ether phospholipids and LysoPCs colocalised with necrotic core, whereas cholesteryl esters, ceramides and PI species were associated with inflammatory regions	Only 6 independent animals; 17 artery sections should not be treated as independent subjects; genetically determined preclinical model; no human validation within the study.

First author, year [Ref.]	Study/model, species and sample size	Arterial bed / plaque phenotype	MALDI modality and principal analytical characteristics	Analyte class	Molecular identification / validation	Principal findings	Principal limitations
**Ntshangase, 2025 [73]**	New Zealand White rabbit model plus human carotid plaques (human n = 3)	Rabbit aortic plaque: neointima and media; human carotid plaque: lipid-necrotic core and fibrous cap/tissue	MALDI-MSI with histology-guided ROI analysis, multivariate modelling and on-tissue fragmentation for selected ions	Lipids, particularly sphingomyelins, phosphatidylcholines and cholesteryl esters	Selected ions underwent MS/MS with optimised collision energy; final identities manually checked against isotopic patterns	Identified 67 differential lipid features between rabbit plaque regions and 199 in human plaque; sphingolipid and glycerophospholipid pathways were disrupted in both species	Human component n = 3; cross-species comparison; rabbit plaque cannot fully reproduce human lesion evolution; exploratory pathway analysis without an independent validation cohort.
**Lohöfer, 2020 [106]**	Female Ldlr−/− mice; 48 mice across the multimodal study, generally n = 3–6/group/time point	Aortic atherosclerosis after 4–20 weeks of high-fat diet	In vivo MRI with Gadofluorine P followed by ex vivo MALDI-MSI and complementary elemental analysis	Exogenous atherosclerosis-affine Gadofluorine imaging agent	Known administered contrast agent; multimodal localisation provides orthogonal confirmation	MALDI-MSI demonstrated spatial localisation of the plaque-affine imaging agent after MRI and linked contrast accumulation to atherosclerotic lesions	Murine model; evaluates an administered imaging probe rather than an endogenous plaque signature; small time-point groups; no human validation.

First author, year [Ref.]	Study/model, species and sample size	Arterial bed / plaque phenotype	MALDI modality and principal analytical characteristics	Analyte class	Molecular identification / validation	Principal findings	Principal limitations
**Shen, 2020 [109]**	WHHL rabbit aortic atherosclerosis with complementary oxidised-LDL and macrophage-cell models; iMScope MS/MS imaging was performed on aortic tissue from one randomly selected WHHL rabbit as a proof-of-concept.	WHHL rabbit aortic lesions of differing sizes	MALDI-TOF-MS plus atmospheric-pressure MALDI imaging microscopy (iMScope); positive ion mode; approximately 10 µm spatial pitch; targeted MS/MS imaging	Oxidised lipids, oxysterols, oxidised cholesteryl esters and lysophosphatidylcholines	Target identities compared against authentic standards and MALDI-MS/MS fragmentation	Visualised characteristic oxidised-lipid and lysoPC distributions within rabbit atherosclerotic lesions and demonstrated lesion-size-associated spatial differences	Proof-of-concept tissue imaging based on one WHHL rabbit; targeted rather than untargeted lipid analysis; animal and cell-model evidence; no human plaque validation.
**Castro-Perez, 2014 [115]**	ApoE-knockout mice fed a high-cholesterol diet (0.15% cholesterol) and orally administered d6-cholesterol over several weeks. The number of animals was not stated in the published abstract; full-text verification was not possible.	Aortic atherosclerotic plaques	MALDI-MSI of ~10 µm tissue sections; matrix applied by sublimation or acoustic-droplet deposition; stable-isotope tracer strategy	Free cholesterol, cholesteryl esters and lysophosphatidylcholines; isotopically labelled cholesterol	Stable-isotope label enabled direct distinction of labelled non-esterified and esterified cholesterol species	Demonstrated plaque-specific spatial accumulation and esterification of d6-cholesterol and mapped LPC and cholesterol-related signals within aortic lesions	Murine stable-isotope tracer study; the animal number was not stated in the accessible abstract and could not be verified from the full text; findings do not directly demonstrate human plaque behaviour or therapeutic efficacy.

First author, year [Ref.]	Study/model, species and sample size	Arterial bed / plaque phenotype	MALDI modality and principal analytical characteristics	Analyte class	Molecular identification / validation	Principal findings	Principal limitations
**Chong, 2023 [116]**	ApoE−/− and Ldlr−/− experimental atherosclerosis models treated with intravenous DHA liposomes; 7–10 mice per group were used for plaque quantification. MALDI-MSI was performed on aortic-root cryosections from ApoE−/− mice after treatment, although the number of animals contributing specifically to the MALDI-MSI analysis was not separately reported.	Aortic/aortic-root experimental plaques; treatment-response setting	MALDI-MSI applied after liposomal DHA treatment to compare plaque lipid profiles with disease stage/treatment	Plaque lipids and treatment-associated lipid-profile changes	Spatial lipid-profile analysis complemented by histology and extensive biological/therapeutic experiments	DHA liposomes accumulated in lesional macrophages, reduced macrophage infiltration and lipid deposition, increased collagen and partly shifted the MALDI lipid profile toward that of earlier-stage plaques	Murine stable-isotope tracer study; preclinical model with limited direct generalisability to human atherosclerotic plaque biology; no human validation.

C. Artificial-plaque, ex vivo and device-related MALDI studies

First author, year [Ref.]	Study/model, species and sample size	Arterial bed / plaque phenotype	MALDI modality and principal analytical characteristics	Analyte class	Molecular identification / validation	Principal findings	Principal limitations
**Razzi, 2022 [112]**	Artificial plaque model implanted ex vivo in porcine coronary arteries; lipid-rich versus lipid-free hydrogel controls	Porcine coronary artery; artificial type-IV-like lipid-rich plaque mounted with a drug-eluting stent	MALDI-MSI used to spatially measure arterial drug distribution behind lipid-rich and lipid-free gelatin/alginate hydrogels	Everolimus / locally delivered drug	Known administered stent drug measured spatially in the arterial wall	Lipid-rich artificial plaque significantly reduced drug transport into the underlying arterial wall compared with lipid-free control hydrogel	Simplified artificial hydrogel cannot reproduce the cellular, inflammatory, necrotic, calcific or structural heterogeneity of human atheroma; ex vivo porcine model; mechanistic drug-transport evidence only.
**Huang, 2012 [121]**	Drug-eluting coronary stents, including stents explanted from porcine coronary arteries	Porcine coronary arterial device model; not spontaneous plaque	MALDI Qq-ToF molecular imaging; coating matrix, laser energy/frequency, spatial resolution and MS parameters systematically optimised	Stent drugs and polymer-associated drug distributions	Identity supported by known drug-eluting-stent compounds and targeted mass-spectrometric detection	Demonstrated MALDI imaging of drug distribution across CYPHER and NEVO drug-eluting stents and feasibility after implantation in porcine coronary arteries	Primarily a device/method-development study; not a model of native human plaque molecular biology; porcine tissue/device healing may not reproduce human atherosclerosis.

D. Complementary human non-imaging MALDI plaque proteomics

First author, year [Ref.]	Study/model, species and sample size	Arterial bed / plaque phenotype	MALDI modality and principal analytical characteristics	Analyte class	Molecular identification / validation	Principal findings	Principal limitations
**Lepedda, 2009 [94]**	Human carotid endarterectomy; n = 48 plaques: 19 stable and 29 unstable	Carotid plaques; unstable lesions defined by thin/fissured cap and/or haemorrhagic, ulcerated or thrombotic features	Non-imaging 2-DE followed by MALDI-MS of proteins extracted from minced plaque	Proteins and proteolytic fragments	Peptide-mass fingerprinting/database identification; fibrinogen-fragment finding additionally supported by Western blotting	Unstable plaques showed lower abundance of SOD3, GST, HSP27, HSP20, annexin A10 and Rho-GDI and greater ferritin light chain, SOD2 and fibrinogen fragment D	Not MSI: tissue extraction/mincing eliminates intraplaque spatial localisation; cross-sectional phenotype; older peptide-mass-fingerprinting workflow; findings do not prospectively predict rupture.
**Liang, 2016 [110]**	Human carotid endarterectomy; n = 6 discovery plaques (3 men/3 women) with n = 20 additional plaques for nLC-MS/MS validation	Internal control, fatty streak, plaque shoulder, plaque centre and fibrous-cap regions	Non-imaging 2-DE/MALDI-TOF on regionally dissected biopsies; additional nLC-MS/MS validation	Proteins	MALDI-TOF peptide-mass fingerprinting identified 52 unique proteins; 41 were independently confirmed by nLC-MS/MS	Eighteen proteins differed among plaque regions; nine showed region-specific changes, particularly involving extracellular-matrix remodelling; sex-associated differences were also reported	Not spatial MSI despite regionally defined biopsy sampling; small MALDI discovery cohort; tissue homogenisation loses microscopic localisation; nLC-MS/MS validation used fewer predefined plaque regions.

Note: Studies are grouped according to experimental model and analytical modality to distinguish direct human plaque/arterial-tissue evidence from animal, mixed-species, artificial-plaque, ex vivo and device-related evidence. Non-imaging MALDI proteomic studies are presented separately because they provide molecular identification from plaque tissue but do not preserve microscopic spatial localisation. Plaque phenotype terminology is retained according to the definitions used in the original studies; consequently, symptomatic/asymptomatic clinical status and stable/unstable histopathological classification are not treated as interchangeable. References [61] and [93] are companion publications arising from the same underlying rabbit experiment and are therefore presented as a single study-level evidence row. DESI, desorption electrospray ionisation; DHB, 2,5-dihydroxybenzoic acid; FT-ICR, Fourier-transform ion cyclotron resonance; MALDI, matrix-assisted laser desorption/ionisation; MSI, mass spectrometry imaging; MS/MS, tandem mass spectrometry; VSMC, vascular smooth-muscle cell.
